# Novel supramolecular artificial light-harvesting systems based on AIE-active macrocycles for efficient white-light photocatalysis in water†

**DOI:** 10.1039/d4sc07689c

**Published:** 2025-02-05

**Authors:** Jun-Cheng Yang, Ke Chen, Guo-Ling Zhang, Chunxuan Qi, Hai-Tao Feng, Ben Zhong Tang

**Affiliations:** a AIE Research Center, Shaanxi Key Laboratory of Phytochemistry, College of Chemistry and Chemical Engineering, Baoji University of Arts and Sciences Baoji Shaanxi 721013 China haitaofeng907@163.com; b School of Science and Engineering, Shenzhen Institute of Aggregate Science and Technology, The Chinese University of Hong Kong Shenzhen 518172 China tangbenz@cuhk.edu.cn

## Abstract

Constructing supramolecular artificial light-harvesting systems (ALHSs) based on the Förster resonance energy transfer (FRET) mechanism provides an optimal platform for understanding natural photosynthesis and simulating natural light-harvesting systems. In the present work, rigid macrocycle K-1 with a nonplanar conformation and aggregation-induced emission (AIE) properties was selected as an energy donor in ALHSs, while the non-cyclic AIEgen K-2 was used for a comparative study. In aqueous solution, an efficient one-step energy-transfer process was established between blue-emitting K-1 and an acceptor (namely PBTB) with orange fluorescence to afford a high energy-transfer efficiency (*Φ*_ET_) of up to 82.6%. Notably, bright white light emission can be readily realized. Moreover, the triad FRET system was fabricated through energy transfer from the AIEgens to PBTB, then further transferring the captured energy to the final red-emitting acceptor (namely as Z1), achieving an efficient two-step sequential energy transfer. When the ratio of K-1/PBTB/Z1 assemblies reached 1000 : 40 : 14, the optimal *Φ*_ET_ was 66.4%. More importantly, it was found that the ALHS based on macrocycle K-1 showed much higher photocatalytic activity for the cross-dehydrogenative coupling (CDC) reaction. Therefore, the flexibility of this novel supramolecular strategy renders the macrocyclic AIEgen a promising candidate to construct efficient ALHSs for photocatalysis.

## Abstract 2

A novel rigid macrocycle with aggregation-induced emission (AIE) properties was facilely synthesized to construct efficient artificial light-harvesting systems for photocatalysis through the Förster resonance energy transfer process.

## Graphical abstract

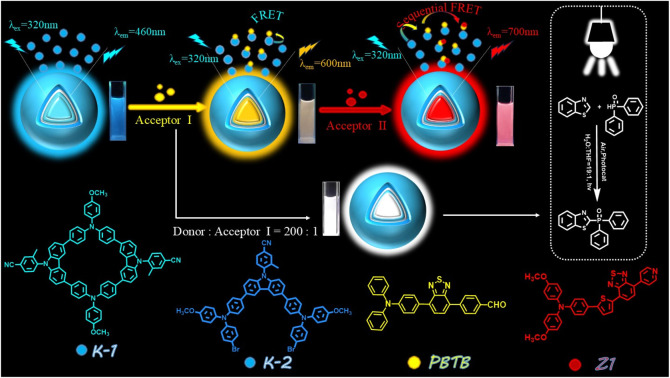

## Introduction

Förster resonance energy transfer (FRET) is a long-range dipole–dipole interaction that occurs between one or more excited fluorophores (donors) and a proximal ground state fluorophore (acceptor).^1–4^ In the distance-dependent photophysical process, energy is transferred between the donor and acceptor in a non-radiative pathway, resulting in fluorescence quenching of the donor and fluorescence enhancement of the acceptor.^5,6^ In general, various fluorescent materials constructed based on the FRET mechanism should have the following prerequisites: (1) an adjacent distance between the donor and acceptor (1–10 nm); (2) an efficient overlap between the donor emission and acceptor absorption; (3) a favorable mutual orientation between the donor emission moment, acceptor absorption moment and their separation vector.^7^ Based on the FRET strategy, many types of materials including organic molecules, polymers, metal–organic frameworks (MOFs), metal nanoparticles (NPs), transition metal complexes, carbon quantum dots (CQDs) and carbon dots (CDs) have been rationally designed over the past decade,^8–13^ which are widely utilized for photocatalysis, anti-counterfeiting, encryption, bioimaging, and fluorescence sensing.^14–18^ It has been an interesting and promising research field to construct artificial light-harvesting systems (LHSs) based on FRET,^19,20^ which not only helps people to gain deeper insights into the photosynthesis process in nature, but also plays a guiding role to mimic natural light-harvesting systems.

Early studies mainly focused on constructing ALHSs through covalent synthesis,^21–23^ however, the synthesis and purification difficulties of these ALHSs hampered their widespread applications, and the low donor/acceptor ratio was not conducive to enhance the efficiency of excitation energy transfer. In the last decade, with increasing interest and further research in artificial LHSs, supramolecular artificial light-harvesting systems based on non-covalent assembly have attracted more attention.^24–30^ To achieve an efficient supramolecular artificial LHS, there are two key factors that we need to take into consideration. First, the energy donor should not only be tightly packed, but also avoid the intramolecular self-quenching of fluorescence resulting from the aggregation-caused quenching (ACQ) effect.^31,32^ Second, the donor/acceptor ratio is advocated to be optimal for energy transfer from multiple donors to one acceptor.^33,34^ These factors can suppress energy loss and improve the excitation energy transfer efficiency (*Φ*_ET_). In contrast to the ACQ effect, fluorescent molecules with aggregation-induced emission (AIE) properties exhibiting strong fluorescence in the aggregated state are promising light-harvesting antennas, which can effectively prevent fluorescence quenching in the FRET process.^35,36^ A commonly employed strategy for the fabrication of supramolecular ALHSs involves the non-covalent encapsulation of AIE luminogens (AIEgens) within supramolecular macrocycles (such as crown ethers,^37^ calix[*n*]arenes,^38–41^ cyclodextrins,^42–45^ cucurbit[*n*]urils,^46–49^ pillar[*n*]arenes,^50,51^ and others^52,53^) and then combining with energy acceptors, which can well satisfy the above prerequisites. Remarkably, due to the confinement effect of the rigid macrocyclic hosts with nonplanar conformations, the intramolecular motions of the guests could be efficiently restricted, thus suppressing the non-radiative decay and favoring the energy transfer. Therefore, rigid macrocyclic molecules, with both nonplanar conformations and AIE properties, have the potential to serve as energy donors, however, the related studies are rarely reported.^54^ As a result, it is desirable to construct novel supramolecular ALHSs based on AIE-active macrocycles to clarify the energy transfer process for photocatalysis.

**Scheme 1:**
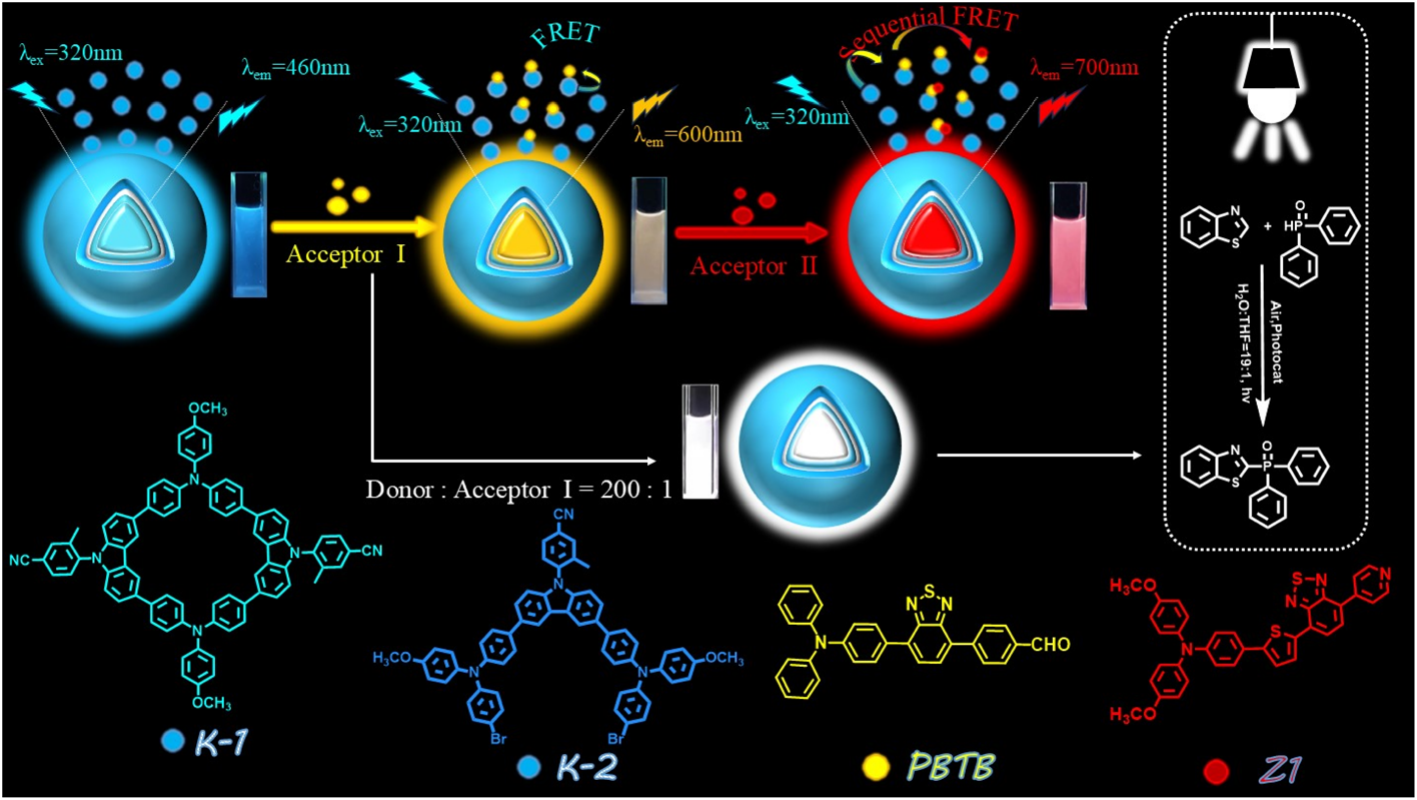
The schematic diagram of the constructed ALHS.

To better simulate the photosynthesis in nature and realize the full utilization of the harvested energy, an effective way is to explore the applications of ALHSs for photocatalysis. Considering the non-planar conformation of triphenylamine (TPA) and the excellent photostability of carbazole (Cz), two blue-emitting energy donors (named as K-1 and K-2) based on TPA and Cz units were synthesized. The introduction of the *o*-methyl group increases the torsion angle between the phenyl ring and carbazole unit, which is beneficial to enhance the aggregated state emission. Therefore, both K-1 and K-2 exhibit obvious AIE properties. The as-prepared macrocycle K-1 can encapsulate guest molecules in the cavity to form supramolecular assemblies avoiding the self-quenching of fluorescence. As a control, the reference molecule K-2 is designed as a noncyclic structure. Then, TPA-based fluorophore PBTB showing orange emission was selected as a relay energy acceptor, and red fluorophore Z1 was used as the terminal acceptor. First, an efficient one-step energy-transfer process was readily established between K-1 and PBTB, with the optimal donor/acceptor (K-1/PBTB) ratio of 1000 : 40, resulting in the *Φ*_ET_ of up to 82.6%. Under the same conditions, the K-2/PBTB assemblies gave a *Φ*_ET_ of 77.9% (Scheme 1). Then, with the introduction of Z1 to the binary system, the calculated *Φ*_ET_ was 66.4% at the ratio of K-1/PBTB/Z1 = 1000 : 40 : 14, while the resultant K-2/PBTB/Z1 assemblies gave a *Φ*_ET_ of 60.4% under the same ratio. In addition, bright white light emission was also observed with CIE coordinates of (0.33, 0.34) at the ratio of K-1/PBTB = 200 : 1 and the ratio of K-2/PBTB = 500 : 3, respectively. More importantly, it was found that the ALHS based on macrocycle K-1 exhibited higher photocatalytic activity for the cross-dehydrogenative coupling (CDC) reactions under white-light in water, with a yield of up to 87%. Furthermore, several organophosphorus compounds were synthesized under optimized photocatalytic conditions. Thus, the tunable feature of the supramolecular strategy makes the macrocyclic AIEgen K-1 a promising candidate for the construction of efficient ALHSs for photocatalysis.

## Results and discussion

As shown in Scheme S1,† the energy donors K-1 and K-2 were rationally designed and facilely synthesized. The carbazole (Cz) derivative 3 was prepared in 78% isolated yield from commercial 3,6-dibromo-9*H*-carbazole 1 and 4-fluoro-3-methylbenzonitrile 2. Next, carbazole derivative 4 was synthesized by Miyaura borylation. The triphenylamine (TPA) derivative 7 was synthesized from 4-methoxyaniline 5 and 1-bromo-4-iodobenzene 6*via* the Ullmann coupling reaction. As expected, K-2 was obtained by the Suzuki coupling reaction of compounds 4 and 7 using Pd(PPh_3_)_4_ as the catalyst. Under the same conditions, the desired K-1 was afforded as a white solid by the reaction of K-2 and intermediate 4. All new compounds were characterized by ^1^H NMR, ^13^C NMR and high resolution mass spectrometry (HRMS). The detailed synthetic procedures and characterization are presented in Scheme S1, Fig. S1–S12 in the ESI.†

After the characterization of the molecular structures of K-1 and K-2, the photophysical properties of the two compounds were investigated using UV-vis absorption spectroscopy and fluorescence emission spectra (Fig. S13†). The absorption spectra of K-1 in THF exhibited an absorption band from 300 to 420 nm with an absorption maximum at around 320 nm (Fig. S13A†). In comparison, the maximum absorption wavelength of the K-2 molecule was 335 nm, showing a slight change from that of K-1 (Fig. S13B†). For a better understanding of the AIE behaviors of K-1 and K-2, their photoluminescence (PL) properties were investigated in THF/H_2_O mixtures with different water fractions (*f*_w_). With the *f*_w_ gradually increasing to 60%, the maximum emission wavelength of K-1 showed an obvious red-shift and the fluorescence intensity decreased significantly, which is the representative twisted intramolecular charge transfer (TICT) process (Fig. S13C†). As the *f*_w_ was further increased from 60% to 90%, the fluorescence intensity started to rise significantly, accompanied by a blue-shift of the maximum emission (Fig. S13D†). This indicated that K-1 exhibits aggregation-induced emission (AIE), meanwhile the aggregates of K-1 were formed in the THF/H_2_O mixture. Similarly, as the *f*_w_ gradually increased to 50%, K-2 also exhibited the TICT effect first with reduced fluorescence and a red-shifted emission band (Fig. S13E†). When the *f*_w_ was further increased, a significant fluorescence enhancement can be observed, which was consistent with the AIE phenomenon (Fig. S13F†).

**Fig. 1:**
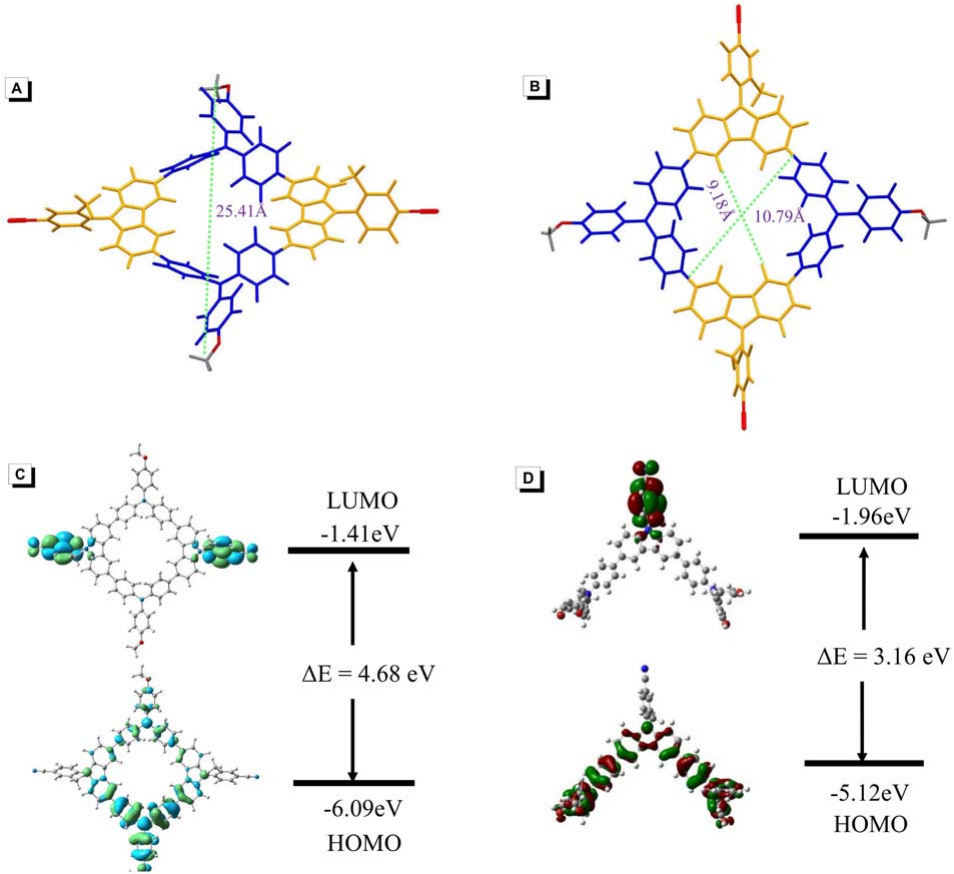
(A) and (B) Optimized chemical structures of K-1. (C) and (D) Molecular orbital and geometry optimizations of K-1 and K-2.

As shown in Fig. 1A, the optimized molecule of K-1 exhibited a highly twisted conformation when viewed from the side, which favors the solid-state emission. The distance between the two *tert*-butyl groups is 25.41 Å. When viewed from the top, it was observed that K-1 has a non-planar cavity (Fig. 1B). The distance between the top C–H bond and the bottom C–H bond was 9.18 Å, and the distance between the left and right phenyl group was 10.79 Å. Subsequently, DFT calculations were performed to collect the structural information on K-1 and K-2. The highest occupied molecular orbital (HOMO) and the lowest unoccupied molecular orbital (LUMO) distributions were depicted, the HOMO was mainly distributed on the triphenylamine (TPA) skeleton, while the LUMO was almost completely situated on the phenylcyano group (Fig. 1C). Due to the strong steric hindrance from the *o*-methyl group,^55,56^ there was a large torsion angle between the phenyl ring and carbazole, resulting in the almost complete separation of the HOMO and LUMO. In comparison with macrocyclic K-1, the HOMO–LUMO gap of branched K-2 was even more narrowed (Fig. 1D), leading to a red-shifted absorption maximum with respect to K-1, which was consistent with the experimental results.

**Fig. 2:**
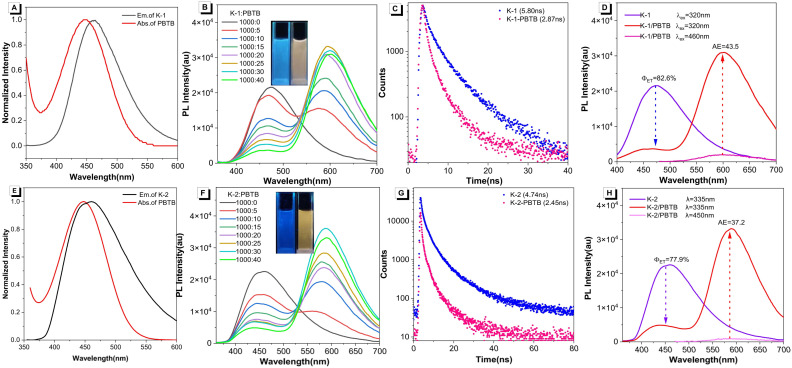
(A) Normalized PL spectrum of K-1 (*λ*_em_ = 460 nm) and the absorption profile of PBTB (*λ*_ex_ = 450 nm). (B) PL spectra of K-1 in water–THF (v/v = 19 : 1) (*λ*_ex_ = 320 nm) with different concentrations of PBTB. (C) Fluorescence decay profiles of the K-1 assembly (blue dots), and K-1/PBTB assemblies (pink dots) (*λ*_em_ = 460 nm). (D) and (H) A representative case showing the calculation principle of *Φ*_ET_ and AE. (E) Normalized PL spectrum of K-2 (*λ*_em_ = 450 nm) and the absorption profile of PBTB (*λ*_ex_ = 450 nm). (F) Fluorescence spectra of K-2 in water–THF (v/v = 19 : 1) (*λ*_ex_ = 335 nm) with different concentrations of PBTB. (G) Fluorescence decay profiles of the K-2 assembly (blue dots) and K-2-PBTB assembly (pink dots) (*λ*_em_ = 450 nm).

Next, the one-step energy transfer process between the two AIEgens and PBTB was investigated. As shown in Fig. 2A, the absorption band of PBTB was well overlapped with the emission of K-1, and therefore the fluorescence dye PBTB was selected as the energy acceptor. Consequently, an efficient energy transfer process from K-1 to PBTB was confirmed in the co-solvents of water/THF by using fluorescence spectroscopy. Upon excitation at 320 nm, the fluorescence spectra of K-1/PBTB assemblies showed a typical Förster resonance energy transfer (FRET) process with one isoemissive point: the fluorescence peak of energy donor K-1 at about 460 nm decreased, while that of energy acceptor PBTB around 600 nm enhanced with the increase of the PBTB concentrations (Fig. 2B). Furthermore, fluorescence lifetime decay experiments at 460 nm were performed to further verify the energy transfer process for the K-1/PBTB assemblies (Fig. 2C and Table S1†). The decay curve of the K-1 was fitted as a double exponential decay with fluorescence lifetimes of *τ*_1_ = 0.94 ns and *τ*_2_ = 3.95 ns. By contrast, the lifetimes in K-1/PBTB assemblies were decreased to *τ*_1_ = 0.70 ns and *τ*_2_ = 2.80 ns under the same conditions, indicating efficient energy-transfer was indeed taken place between K-1 and PBTB. The above results suggested that the artificial LHS based on the K-1/PBTB system was successfully constructed. To quantitatively evaluate the performance of this prepared LHS, the energy-transfer efficiency and antenna effect (AE) were calculated, which were widely used as empirical parameters for the evaluation of light-harvesting ability. In this system, the *Φ*_ET_ was calculated as 82.6% (eqn (S1)†), and the antenna effect was calculated to be 13.1 (eqn (S2)†) at a donor/acceptor ratio of 1000 : 40 (Fig. 2D and Table S2†). In addition, the energy-transfer efficiency can be gradually increased with the K-1/PBTB ratio from 1000 : 5 to 1000 : 40 (Table S2†). Moreover, the emission spectrum of K-2 effectively overlapped with the absorption spectra of the acceptor PBTB (Fig. 2E), suggesting the possibility of energy transfer from K-2 to PBTB. As depicted in Fig. 2F, with addition of PBTB to K-2, the PL intensity of PBTB (acceptor) around 600 nm increased along with the decrease of K-2 (donor) emission at approximately 450 nm when excited at 335 nm in water–THF (19 : 1; v/v). In addition, the fluorescence lifetime decay experiments of the light-harvesting system monitored at 450 nm were also performed (Fig. 2G). The results showed double exponential decay with fluorescence lifetimes of 0.60 and 3.11 ns for K-2, which decreased to 0.45 and 2.30 ns after the addition of PBTB, indicating the energy transfer from K-2 to PBTB (Table S3†). Therefore, the above data clearly demonstrated the efficient energy transfer from K-2 to PBTB. The energy transfer efficiency was estimated to be 77.9% with a molar ratio of donor/acceptor of 1000 : 40 (Fig. 2H and Table S4†), and the antenna effect was calculated to be 37.2 at such donor/acceptor ratio, indicating that the K-2/PBTB could also function as an efficient light harvesting antenna in an aqueous environment.

**Fig. 3:**
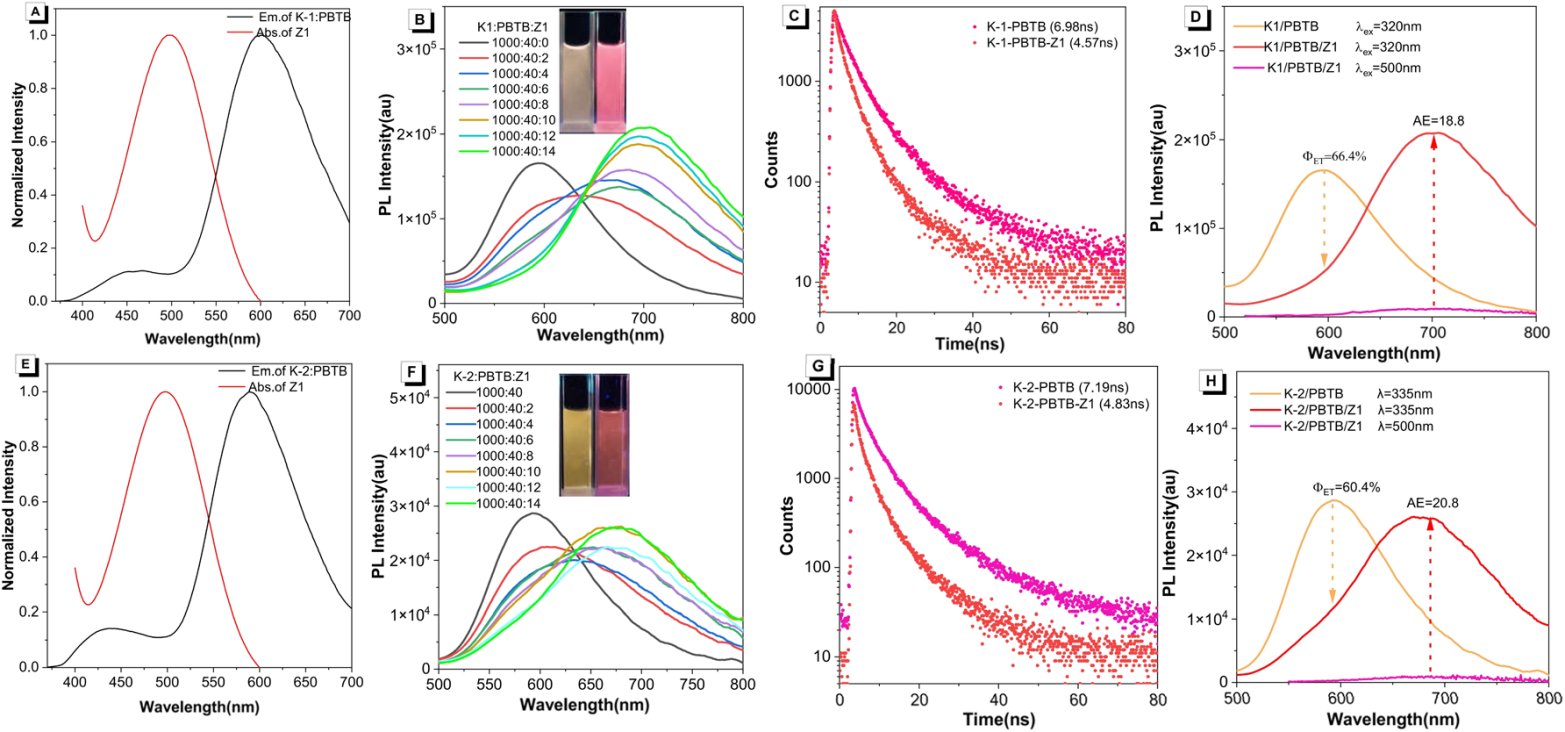
(A) Normalized absorption and emission spectra of K-1/PBTB = 1000 : 40 (*λ*_em_ = 600 nm) and Z1 (*λ*_ex_ = 500 nm). (B) Fluorescence spectra of K-1/PBTB = 1000 : 40 with different concentrations of Z1. (C) Fluorescence decay profiles of the K-1/PBTB assembly (pink dots) and K-1/PBTB/Z1 assembly (red dots) (*λ*_em_ = 600 nm). (D) and (H) A representative case showing the calculation principle of *Φ*_ET_ and AE. (E) Normalized absorption and emission spectra of K-2/PBTB = 1000 : 40 (*λ*_ex_ = 590 nm) and Z1 (*λ*_ex_ = 500 nm). (F) Fluorescence spectra of K-2/PBTB = 1000 : 40 with different concentrations of Z1. (G) Fluorescence decay profiles of the K-2/PBTB assembly (pink dots) and K-2/PBTB/Z1 assembly (red dots) (*λ*_em_ = 590 nm).

Given that the photosynthetic light-harvesting system in nature contains multi-step sequential energy transfer with multichromophoric assemblies,^57–59^ multi-step sequential energy transfer rather than simple one-step energy transfer is more meaningful for ALHSs. Therefore, based on the efficient one-step energy transfer achieved by K-1/PBTB, we further investigated the two-step sequential energy transfer process. As depicted in Fig. 3A, the absorption region of the triphenylamine-based organic dye Z1 partially overlapped with the emission region of the K-1/PBTB complex, making it possible to realize the two-step FRET process. As shown in Fig. 3B, when excited at 320 nm, the emission intensity of K-1/PBTB at 600 nm showed a marked decrease with the gradual addition of Z1. Meanwhile, the maximum emission wavelength red-shifted from 600 to 700 nm, accompanied by a substantial increase in fluorescence intensity. The fluorescence decay experiments showed a decline of the fluorescence lifetime from the K-1/PBTB assemblies (*τ*_1_ = 3.63 ns, *τ*_2_ = 11.22 ns) to the K-1/PBTB/Z1 assemblies (*τ*_1_ = 2.36 ns, *τ*_2_ = 7.96 ns) when monitored at 600 nm (Fig. 3C and Table S5†). When the ratio of K-1/PBTB/Z1 reached 1000 : 40 : 14, the *Φ*_ET_ was calculated to be 66.4% and the AE was 18.2 (Fig. 3D). Based on the prerequisites for efficient light-harvesting, we speculated that the relatively small overlap between the absorption spectra of Z1 and the emission spectra of PBTB could result in reduced energy transfer efficiency. In general, the above results indicate that K-1/PBTB/Z1 could realize an efficient two-step sequential energy transfer process. Encouraged by the above light-harvesting process of K-1/PBTB/Z1, K-2/PBTB complexes and Z1 were also suitable for undergoing of two-step energy transfer due to the partial overlap between the fluorescence emission band of the K-2/PBTB complex and the UV-visible absorption band of Z1 (Fig. 3E). As presented in Fig. 3F, with the increment of Z1 concentration, the fluorescent intensity of energy donor K-2/PBTB complexes at 590 nm emission gradually decreased and the intensity of acceptor Z1 at 680 nm emission obviously increased. Simultaneously, the fluorescent color of the K-2/PBTB/Z1 complex exhibited a significant change from orange to red (inset of Fig. 3F). In addition, compared to the *Φ*_ET_ of K-1/PBTB/Z1, the result of the K-2/PBTB/Z1 at the same ratio was 60.4% (Fig. 3H), exhibiting a small decline. In contrast, the AE value of K-2/PBTB/Z1 was 20.8. Moreover, fluorescence decay profiles were also obtained in the K-2/PBTB/Z1 system (Fig. 3G). Compared with the lifetimes of K-2/PBTB, the result of K-2/PBTB/Z1 at 590 nm exhibited a significant decrease (Table S6†). In general, the transfer efficiency of the constructed two-step energy transfer was reduced to some extent (Tables S7 and S8†). In a control experiment, free PBTB or Z1 without energy donors (K-1 or K-2) showed almost no fluorescence upon excitation, indicating efficient energy transfer from the energy donor to acceptor (Fig. S14†). Subsequently, the morphologies of the above-mentioned individual molecules and ALHSs were investigated by scanning electron microscopy (SEM). The results showed that these nanoparticles appeared to have concave or almost regular spherical morphology (Fig. S15 and S16†). Therefore, the sizes of the formed aggregates after assembly were further studied by dynamic light scattering (DLS) experiments. The results of DLS showed that spherical nanoparticles with larger average particle size were formed after the acceptors were mixed with donors (Fig. S17 and S18†), indicating extensive interactions and co-assembly processes between molecules. In addition, obvious Tyndall effects could be observed in all solutions (inset of Fig. S17 and S18†), which also indicated that a large number of supramolecular nanoparticles were formed.

**Fig. 4:**
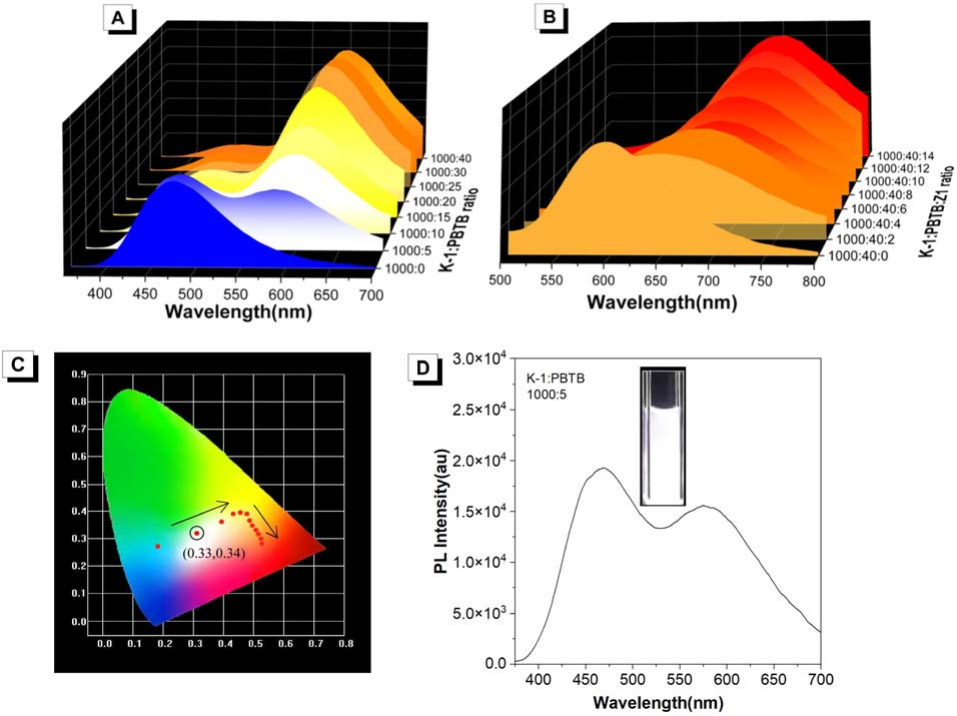
(A) The PL spectra of K-1 in water with different concentrations of PBTB. (B). The PL spectra of K-1/PBTB in water with different concentrations of Z1. (C) CIE chromaticity coordinates of K-1/PBTB with different concentrations of Z1. (D) PL spectrum of the white-light emission coordinate (D/A = 200 : 1), inset: photograph of the white-light emission.

White organic light-emitting materials are of significance because of their potential applications in light emitting devices and display media.^60–62^ Generally, white light emission can be achieved by a combination of the three primary (red, green, and blue) or two complementary colors (*e.g.* orange and blue) according to the Commission Internationale de L'Eclairage (CIE) chromaticity diagram. As the ratio of PBTB increased, the fluorescence color of the K-1/PBTB system changed from blue to orange (Fig. 4A), indicating that white light emission can be realized by tuning the ratio of K-1 and PBTB. In contrast, it is speculated that the K-1/PBTB/Z1 system failed to achieve white light emission due to its color change from orange to red as the ratio of Z1 increased (Fig. 4B). As expected, bright white light emission can be successfully realized with CIE coordinates of (0.33, 0.34) at the ratio of K-1/PBTB = 200 : 1 (Fig. 4C and D). When the energy donor was replaced by K-2, similar results were obtained in the corresponding ALHSs (Fig. S19†).

Due to the wide application of organophosphorus compounds in pharmaceuticals, pesticides, flame retardants, ligands and so on, relevant research reports are of great significance.^63–66^ Therefore, photocatalytic C–P bond formation has attracted great attention among chemists,^67,68^ especially the reports of ALHSs as photocatalysts.^69,70^ We then investigated the possibility of the above ALHSs and individual fluorescent dyes as photocatalysts in cross-dehydrogenative coupling (CDC) reactions, and optimized the conditions of photocatalytic C–P bond formation (Tables 1, 2 and Fig. S20†).

**Table 1:** Comparison result using ALHSs as photocatalysts for the CDC reaction of benzothiazole 8a and diphenylphosphine oxide 9

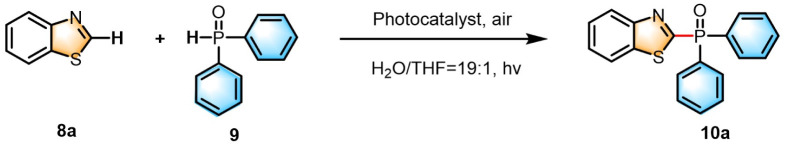		
Entry^a^	Photocatalyst	Yield (%)
1	K-1	52
2	K-2	29
3	PBTB	28
4	Z1	12
5	K-1/PBTB = 200 : 1	87
6^b^	K-1/PBTB = 200 : 1	78
7^c^	K-1/PBTB = 200 : 1	65
8^d^	K-1/PBTB = 200 : 1	Trace
9^e^	No	8
10^f^	K-1/PBTB = 200 : 1	n. r.
11	K-1/PBTB = 1000 : 40	72
12	K-1/PBTB/Z1 = 1000 : 40 : 14	70
13	K-2/PBTB = 500 : 3	35
14	K-2/PBTB = 1000 : 40	36
15	K-2/PBTB/Z1 = 1000 : 40 : 14	42

a Reaction conditions: benzothiazole (0.2 mmol), the photocatalyst (5% mmol), diphenylphosphine oxide (1.2 mmol, 6 equiv.), H_2_O (1.9 mL), THF (0.1 mL) 30 W white LED, and under air at room temperature for 24 h.

b 3% photocatalyst.

c Diphenylphosphine oxide (0.6 mmol, 3 equiv.).

d Under N_2_.

e No PC.

f No light.

In a mixed solution of H_2_O–THF (19 : 1; v/v), benzothiazole 8a was selected as the model substrate for the reaction with diphenylphosphine oxide 9, and the effect of the photocatalysts was first investigated (Table 1). The experimental results revealed that the target product 10a was formed in poor to moderate yields, when K-1, K-2, PBTB, and Z1 were employed as photocatalysts (Table 1, entries 1–4). The TLC analysis revealed that 8a was not fully transformed in all the above reactions. Notably, the reaction yield exhibited an impressive enhancement, reaching 87% when the K-1/PBTB ALHS was used as the catalyst (Table 1, entry 5). Moreover, the solution emitted white light at the ratio of K-1/PBTB of 200 : 1. When the amount of catalyst was reduced to 3 mol%, the yield was slightly reduced (Table 1, entry 6). In addition, the improved equivalent of 9 failed to increase the yield, and the product 10a was obtained with a moderate yield of 65% (Table 1, entry 7). Subsequently, a series of control experiments were performed. As expected, a trace amount of the product was observed in a N_2_ atmosphere (Table 1, entry 8), and a small amount of product (8%) was detected in the absence of photocatalysts (Table 1, entry 9). Unsurprisingly, this reaction did not occur in the absence of light (Table 1, entry 10). Since the maximum *Φ*_ET_ occurred when the K-1/PBTB was about 1000 : 40, the reaction was conducted under this ratio of donor/acceptor. The result showed that 72% yield of 10a can be obtained (Table 1, entry 11). For the same reason, the reaction was performed at the K-1/PBTB/Z1 ratio of 1000 : 40 : 14, and 10a was isolated in 70% yield (Table 1, entry 12). Unfortunately, the reactions resulted in lower yields at different ratios of donor and acceptor when the energy donor was replaced with K-2 (Table 1, entries 13–15). In addition, when the reaction was carried out in various organic solvents, chloroform afforded a high yield of 90%. As most natural photocatalysis occurs in aqueous medium, we selected water as the reaction solvent. Moreover, other organic solvents only gave moderate yields, or trace amounts of the desired product can be formed (Table 2).

**Table 2:** The CDC reaction of benzothiazole 8a and diphenylphosphine oxide 9 in organic solvents

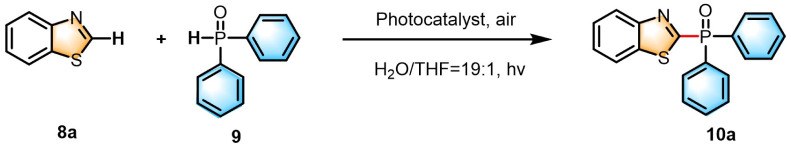			
Entry^a^	Photocatalyst	Solvent	Yield (%)
1	K-1/PBTB = 200 : 1	MeOH	n. r.
2	K-1/PBTB = 200 : 1	EtOH	n. r.
3	K-1/PBTB = 200 : 1	CH_3_CN	Trace
4	K-1/PBTB = 200 : 1	THF	18
5	K-1/PBTB = 200 : 1	CHCl_3_	90
6	K-1	CHCl_3_	88
7	K-1/PBTB = 200 : 1	CH_2_Cl_2_	40
8	K-1/PBTB = 200 : 1	EtOAc	12

a Reaction conditions: benzothiazole (0.2 mmol), the photocatalyst (5% mmol), diphenylphosphine oxide (1.2 mmol, 6 equiv.), solvent (2 mL), 30 W white LED, and under air at room temperature for 24 h.

**Scheme 2:**
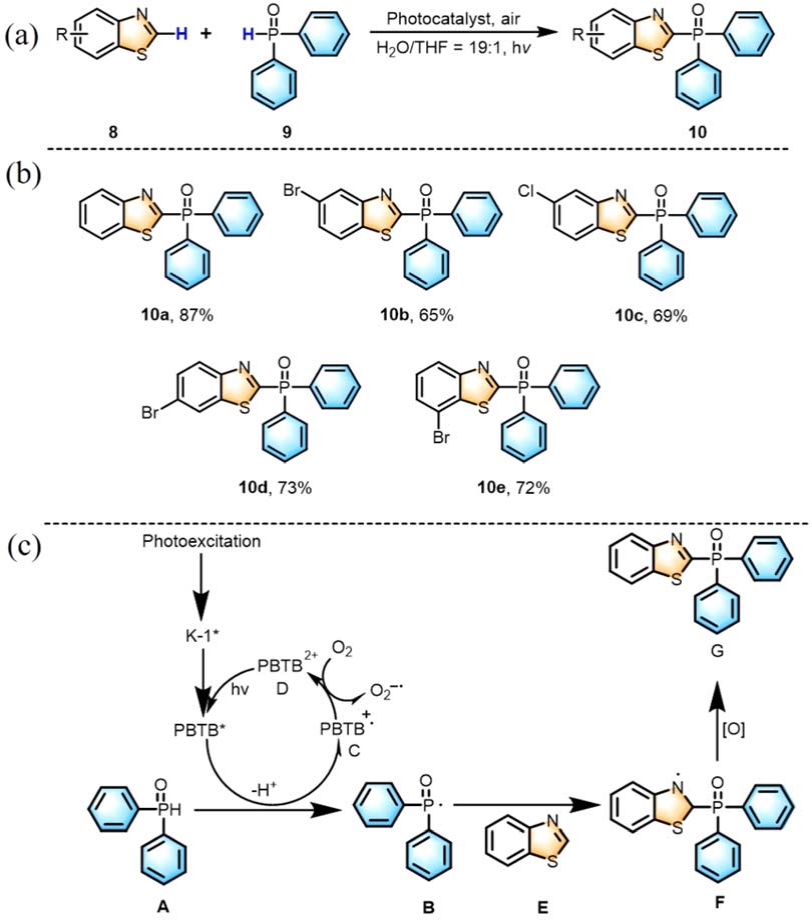
(a) Visible-light-induced C(sp^2^)–P bond formation catalyzed by K-1/PBTB. (b) Substrate scope of the C(sp^2^)–P bond formation reaction. (c) Proposed mechanism of the C(sp^2^)–P bond formation reaction.

Furthermore, the reaction of different benzothiazole derivatives with diphenylphosphine oxide yielded the desired products (10a–10e) in moderate to good yields (65–87%) (Fig. S21–S35†). We proposed a mechanism for the construction of the C–P bond. The excited state K-1* interacted with A and deprotonated to give a radical B and a radical cation C. Then, the radical cation C was oxidized by O_2_ and regenerated ground state D to complete the photocatalytic cycle. Meanwhile, radical species B attacked benzothiazole E to furnish radical intermediate F, which underwent rearomatization by oxidation and deprotonation to afford the final product (Scheme 2).

## Conclusions

In summary, novel supramolecular ALHSs based on AIE-active macrocycle K-1 were constructed, which successfully achieved one-step and two-step FRET processes. Thanks to the typical AIE properties, undesirable fluorescence quenching is effectively avoided during the FRET process. By contrast with the non-cyclic AIEgen K-2, the ALHSs based on rigid macrocyclic molecule K-1 exhibited higher energy transfer efficiency, which was calculated to be 82.6% for the K-1/PBTB system and 66.4% for the K-1/PBTB/Z1 system, respectively. Bright white light emission can be successfully achieved at the ratio of K-1/PBTB = 200 : 1 and K-2/PBTB = 500 : 3 with CIE coordinates of (0.33, 0.34), respectively. More importantly, the ALHS based on macrocycle K-1 exhibited higher photocatalytic activity for the cross-dehydrogenative coupling (CDC) reaction under white-light in water, with yields of up to 87%. Thus, the flexibility of this novel supramolecular strategy makes macrocyclic AIEgen K-1 a promising candidate for the construction of efficient ALHSs for photocatalysis. Compared with the existing approaches to realize ALHSs, this work provided an appealing strategy to construct novel supramolecular ALHSs based on AIE-active macrocycles for photocatalysis.

## Data availability

The authors confirm that the data supporting the findings of this study are available within the article and its ESI.†

## Author contributions

Jun-Cheng Yang and Ke Chen contributed equally to this work. Jun-Cheng Yang: conceptualization, methodology, writing – original draft. Ke Chen: methodology, investigation. Guo-Ling Zhang: investigation. Chunxuan Qi: investigation. Hai-Tao Feng: conceptualization, funding acquisition, writing – review & editing. Ben Zhong Tang: conceptualization, writing – review & editing.

## Conflicts of interest

There are no conflicts to declare.

## Supplementary Material

SC-OLF-D4SC07689C-s001
