# Supplementary material for: Novel supramolecular artificial light-harvesting systems based on AIE-active macrocycles for efficient white-light photocatalysis in water
Source: Chem Sci. 2025 Feb 5;16(11):4741–8. doi: 10.1039/d4sc07689c (PMC11826479; doi:10.1039/d4sc07689c)
Supplement: SC-016-D4SC07689C-s001 [file SC-016-D4SC07689C-s001.pdf]

Supplementary Information (SI) for Chemical Science.
This journal is © The Royal Society of Chemistry 2025

Supporting Information

Novel Supramolecular Artificial Light-Harvesting Systems Based on AIE-active Macrocycle for Efficient White-Light Photocatalysis in Water

Jun-Cheng Yang,‡[a] Ke Chen,‡[a] Guo-Ling Zhang,[a] Chunxuan Qi,[a] Hai-Tao Feng,[a] and Ben Zhong Tang*[b]*

J.-C. Yang, K. Chen, G.-L. Zhang, C. Qi, Prof. H.-T. Feng
AIE Research Center, Shaanxi Key Laboratory of Phytochemistry, College of Chemistry and Chemical Engineering, Baoji University of Arts and Sciences
Baoji, Shaanxi 721013, China
E-mail: haitaofeng907@163.com

Prof. B. Z. Tang
School of Science and Engineering, Shenzhen Institute of Aggregate Science and Technology, The Chinese University of Hong Kong
Shenzhen 518172, China
E-mail: tangbenz@cuhk.edu.cn

1. General information

Materials: All reagents and solvents were chemical pure (CP) grade or analytical reagent (AR) grade and were used as received unless otherwise indicated. Column chromatography was performed with silica gel (200-300 mesh) produced by Qingdao Marine Chemical Factory, Qingdao (China). All yields were given as isolated yields.

Measurements: ^{1}H NMR and ^{13}C NMR spectra were obtained by an Agilent NMR Systems 400 MHz NMR Spectrometer at 298 K in $CDCl_3$. High-resolution mass spectra (HRMS) were measured by an AB SCIEX 4600 mass spectrometer. Absorption spectra were recorded on a Shimadzu UV-2550 UV-Vis spectrophotometer. Fluorescence spectra were collected on a HORIBA FLOUROMAX-4 fluorophotometer at 298 K. The surface morphologies of the samples were analyzed using scanning electron microscope (SEM, FEI Quanta FEG 250). The luminescence lifetimes were measured on an Edinburgh FLS 1000 fluorescence spectrometer operating intime-correlated single-photon counting (TCSPC) mode.

2. General procedure for the synthesis of K-1 and K-2

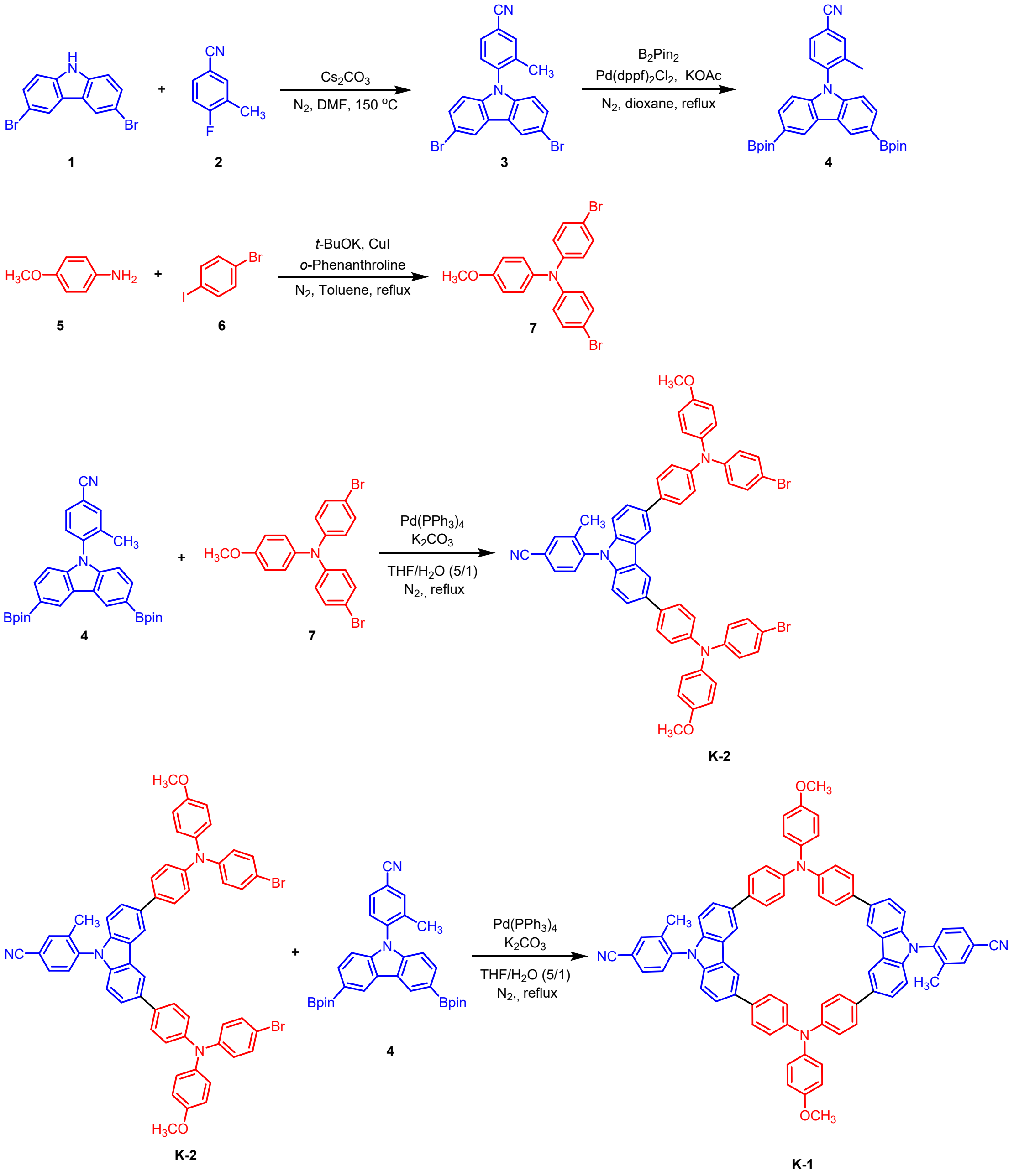

Scheme S1. Synthetic route of **K-1** and **K-2**.

Synthesis of 4-(3,6-dibromo-9H-carbazol-9-yl)-3-methylbenzonitrile (3)

Into a 250 mL three-necked flask was added compound **1** (4.87 g, 15 mmol), Cs_2CO_3 (14.66 g, 45 mmol), compound **2** (2.43 g,18 mmol) and DMF (80 mL) under nitrogen atmosphere, followed by stirring for overnight at 150 °C. After that, the mixture was poured into water (200 mL) and extracted with ethyl acetate (3×100 mL). The combined organic phase was washed with brine, dried over anhydrous Na_2SO_4 and then concentrated in *vacuo*. The residue was purified by silica gel column chromatography (petroleum ether/ethyl acetate = 10:1 to 5:1). White solid of **3** was isolated in 78% yield. ^{1}H NMR (400 MHz, $CDCl_3$) δ = 8.22 (d, J = 1.6 Hz, 2H), 7.80 (s, 1H), 7.73 (dd, J = 8.0, 1.6 Hz, 1H), 7.51 (dd, J = 8.8, 2.0 Hz, 1H), 7.47 (d, J = 8.0 Hz, 1H), 6.87 (d, J = 8.8 Hz, 2H) 2.00 (s, 3H). ^{13}C NMR (100 MHz, $CDCl_3$) δ = 139.52, 139.44, 138.81, 135.55, 131.32, 130.05,

129.77, 123.99, 123.56, 117.96, 113.57, 113.25, 111.16, 17.52.

Synthesis of 4-(3,6-bis(4,4,5,5-tetramethyl-1,3,2-dioxaborolan-2-yl)-9*H*-carbazol-9-yl)-3-methylbenzonitrile (4)

Compound **3** (2.2 g, 5 mmol) was added to a mixture of B_2pin_2 (3.8 g, 15 mmol), Pd(dppf)Cl_2 (0.37 g, 0.5 mmol), and potassium *tert*-butoxide (1.47 g, 15 mmol) in 1,4-dioxane (40 mL) at room temperature under nitrogen atmosphere. The mixture was stirred for overnight at 85 °C, and then allowed to cool to room temperature. After that, the solvent was removed under reduced pressure and the residue was purified by column chromatography on silica gel using petroleum ether/ethyl acetate as eluent (petroleum ether/ethyl acetate = 10:1) to give the desired product **4** as a white solid (75% yield). ^{1}H NMR (400 MHz, $CDCl_3$) δ = 8.73 (s, 2H), 7.85 (dd, *J* = 8.4, 1.2 Hz, 2H), 7.80 (s, 1H), 7.74-7.71 (m, 1H), 7.51 (d, *J* = 8.0 Hz, 1H), 6.97 (d, *J* = 8.4 Hz, 2H), 2.01 (s, 3H), 1.39 (s, 24H). ^{13}C NMR (100 MHz, $CDCl_3$) δ = 142.62, 140.25, 138.93, 135.45, 132.62, 131.20, 130.19, 128.21, 123.26, 118.21, 112.83, 108.86, 83.70, 24.94, 17.58. ESI$^+$ HRMS m/z calcd for $C_{32}H_{36}B_2N_2O_4$ 535.2934 [M+H]$^+$, found 535.2948.

Synthesis of 4-bromo-*N*-(4-bromophenyl)-*N*-(4-methoxyphenyl)aniline (7)

Into a 100 mL three-necked flask was added **5** (0.49 g, 4 mmol), **6** (2.8 g, 10 mmol), CuI (0.023 g, 0.12 mmol), *o*-Phenanthroline (0.022 g, 0.12 mmol) and 15 mL toluene under nitrogen atmosphere, followed by stirring for overnight at 110 °C. After cooled to room temperature, mixture was filtered with suction and the filtrate was collected. Then, the solvent was removed under reduced pressure and the residue was purified by column chromatography on silica using petroleum ether as the eluent to give **7** as white solid (35% yield). ^{1}H NMR (400 MHz, $CDCl_3$) δ = 7.30 (d, *J* = 8.8 Hz, 4H), 7.02 (d, *J* = 8.8 Hz, 2H), 6.89 (d, *J* = 8.8 Hz, 4H), 6.85 (d, *J* = 9.2 Hz, 2H), 3.80 (s, 3H). ^{13}C NMR (100 MHz, $CDCl_3$) δ = 156.71, 146.79, 139.70, 132.14, 127.38, 124.25, 115.01, 114.52, 55.48.

Synthesis of K-2

4 (0.13 g, 0.25 mmol), **7** (0.23 g, 0.53 mmol), K_2CO_3 (0.28 g, 2 mmol) and Pd(PPh_3)$_4$ (0.03 g, 0.025 mmol) were added into a 250 mL three-necked flask under nitrogen atmosphere, and then a mixture of THF (50 mL) and H_2O (10 mL) were added. Then, the solution was cooled to room temperature and quenched with water. Afterward, the mixture was extracted with dichloromethane (3×50 mL). The combined organic layer was washed with brine, dried over Na_2SO_4 and then the solvent was removed under reduced pressure. The residue was purified by column chromatography on silica gel (petroleum ether/ethyl acetate = 20:1) to provide **K-2** as a white solid in 55% yield. ^{1}H NMR (400 MHz, $CDCl_3$) δ = 8.35 (d, *J* = 1.6 Hz, 2H), 7.82 (d, *J* = 1.2 Hz, 1H), 7.74 (dd, *J* = 8.0, 1.6 Hz, 1H), 7.64 (d, *J* = 1.6 Hz, 1H), 7.61 (d, *J* = 2.0 Hz, 1H), 7.58-7.56 (m, 4H), 7.55 (s, 1H), 7.32 (d, *J* = 8.8 Hz, 4H), 7.15-7.11 (m, 8H), 7.05 (d, *J* = 8.4 Hz, 2H), 6.97 (d, *J* = 9.2 Hz, 4H), 6.88 (d, *J* = 9.2 Hz, 4H), 3.82 (s, 6H), 2.12 (s, 3H). ^{13}C NMR (100 MHz, $CDCl_3$) δ = 156.54, 147.23, 146.50, 140.53, 140.18, 140.13, 138.85, 135.68, 135.48, 133.54, 132.02, 131.17, 130.14, 127.96, 127.38, 125.57, 124.10, 123.98, 123.45, 118.60, 118.25, 114.95, 113.93, 112.64, 109.89, 55.50, 17.80. ESI$^+$ HRMS m/z calcd for $C_{58}H_{42}Br_2N_4O_2$ 986.1654 [M+H]$^+$, found 987.1739.

Synthesis of K-1

Into a 250 mL three-necked flask was added **K-2** (1.2 g, 2.4 mmol), **4** (0.65 g, 2.4 mmol), K_2CO_3 (1.35 g, 9.8 mmol) and Pd(PPh_3)$_4$ (0.27 g, 0.24 mmol) under nitrogen atmosphere, followed by adding a mixture of THF (50 mL) and H_2O (10 mL). Then, the reaction was stirred for overnight at 85 °C and then allowed to cool to room temperature. After that, the solution was quenched with water and extracted with dichloromethane (3×50 mL). The combined organic phase was washed

with brine and dried over Na_2SO_4, followed by removal of the solvent in *vacuo*. The residue was purified by column chromatography on silica gel using petroleum ether/ethyl acetate (4:1) as the eluent to give the desired **K-1** as a white solid in 8% yield. ^{1}H NMR (400 MHz, DMSO-d_6) δ = 8.40 (s, 2H), 8.08 (s, 2H), 7.92 (d, J = 7.6 Hz, 2H), 7.67 (d, J = 8.4 Hz, 4H), 7.64-7.60 (m, 6H), 7.39 (d, J = 8.8 Hz, 4H), 7.08 (t, J = 8.8 Hz, 8H), 7.01-6.91 (m, 8H), 6.89-6.84 (m, 6H), 3.76 (s, 6H), 1.98 (s, 6H). ^{13}C NMR (100 MHz, DMSO-d_6) δ = 156.38, 151.85, 147.01, 145.64, 140.21, 139.72, 139.25, 138.06, 135.59, 135.24, 134.57, 131.94, 131.58, 131.42, 129.99, 127.68, 127.54, 124.86, 123.71, 123.39, 123.33, 123.14, 118.43, 118.26, 115.71, 115.18, 112.71, 111.20, 110.21, 109.83, 105.77, 55.24, 17.12. ESI$^+$ HRMS m/z calcd for $C_{78}H_{54}N_6O_2$ 1106.4308 $[M+2H]^+/2$, found 554.2889.

Z1 and **PBTB** are known compounds, which were synthesized according to previous reports [S1,S2].

3. Characteristic spectra

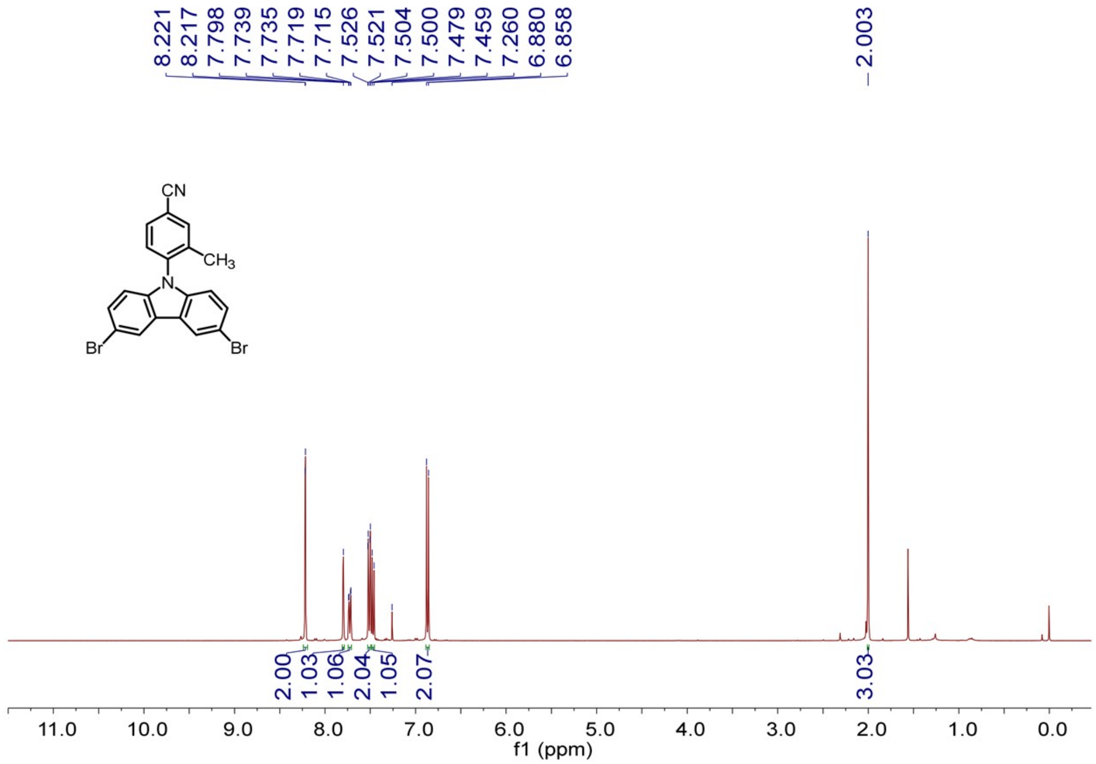

Fig. S1 ^{1}H NMR spectra of **3** (in $CDCl_3$).

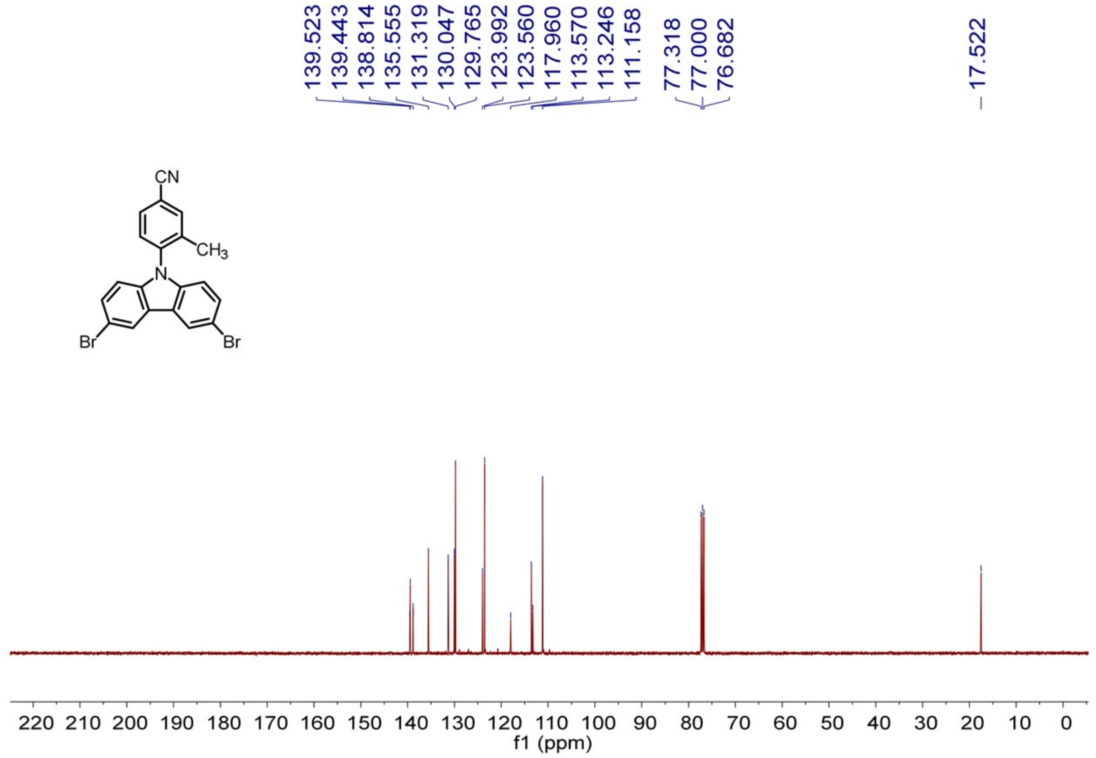

Fig. S2 ^{13}C NMR spectra of **3** (in $CDCl_3$).

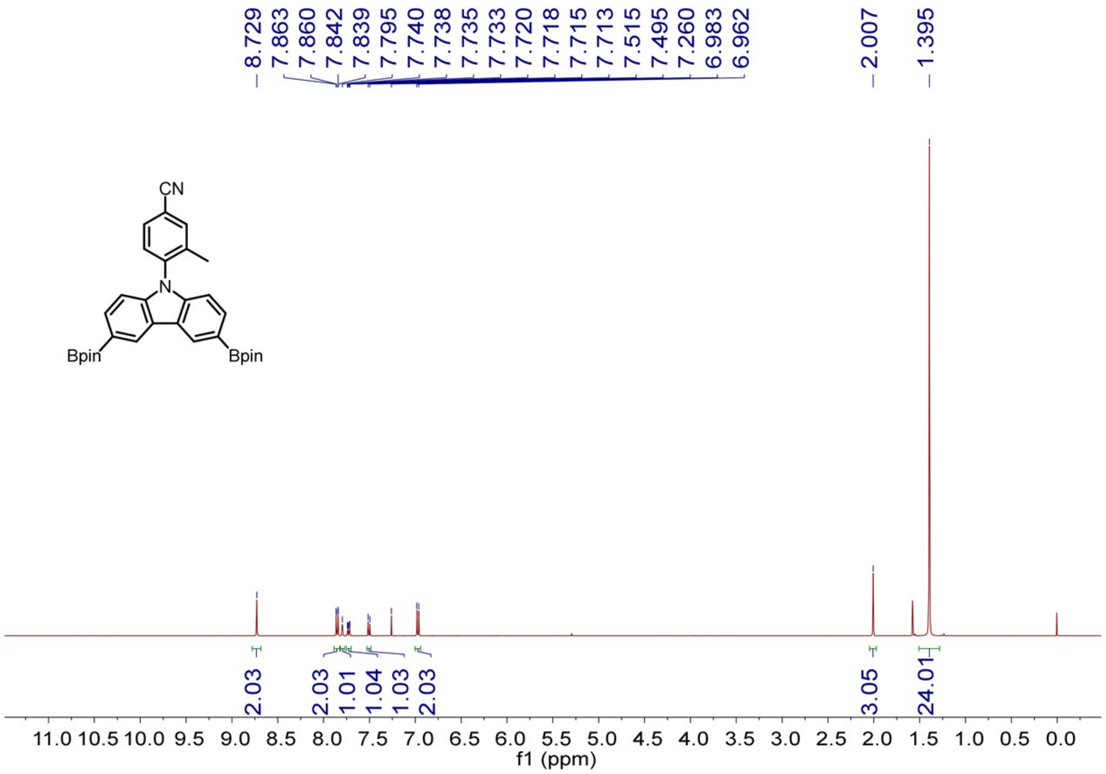

Fig. S3 ^{1}H NMR spectra of **4** (in $CDCl_3$).

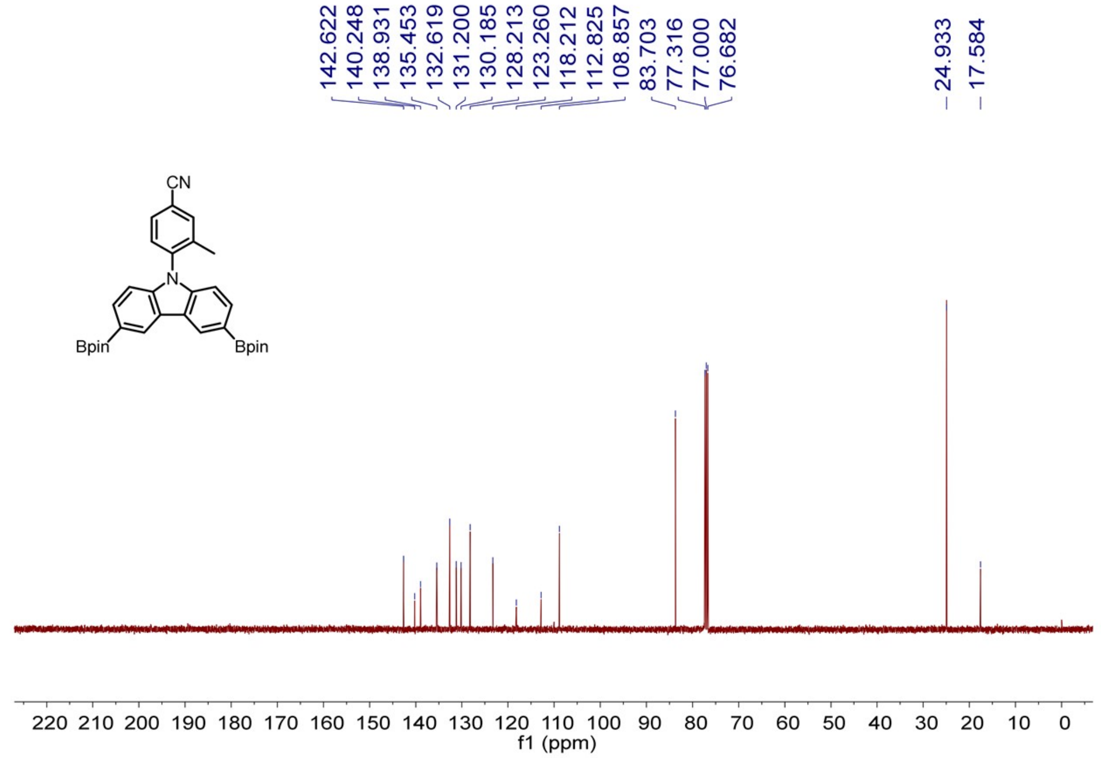

Fig. S4 ^{13}C NMR spectra of **4** (in $CDCl_3$).

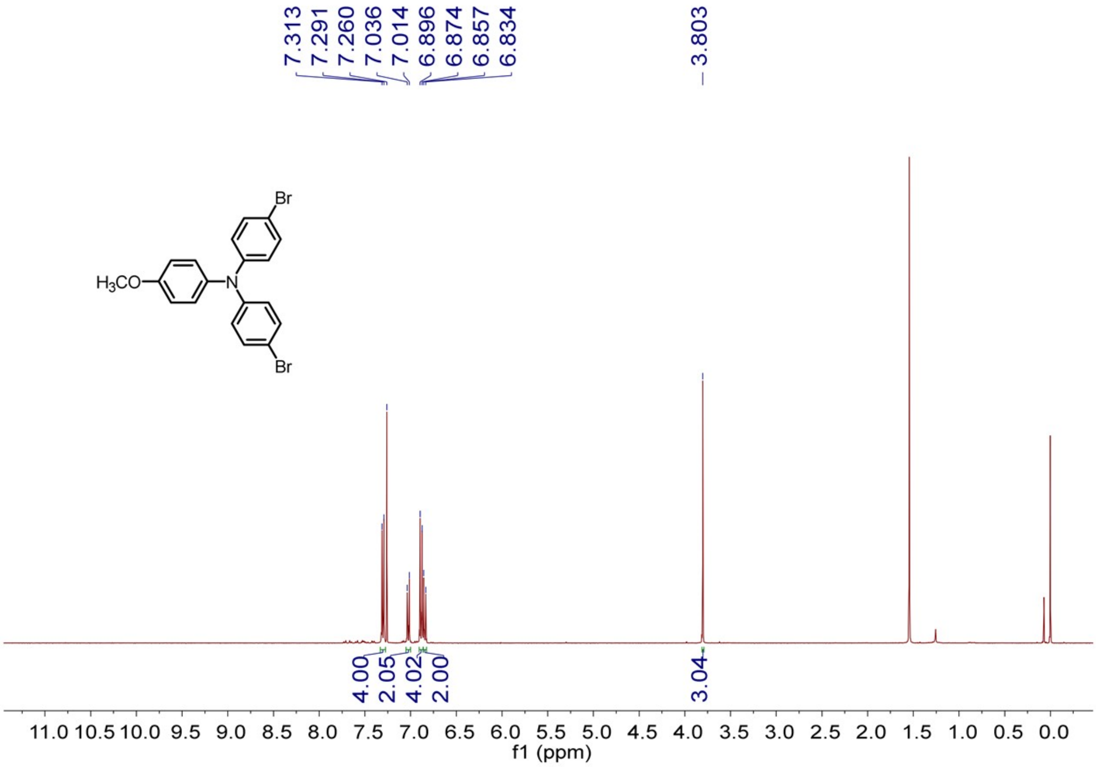

Fig. S5 ^{1}H NMR spectra of **7** (in $CDCl_3$).

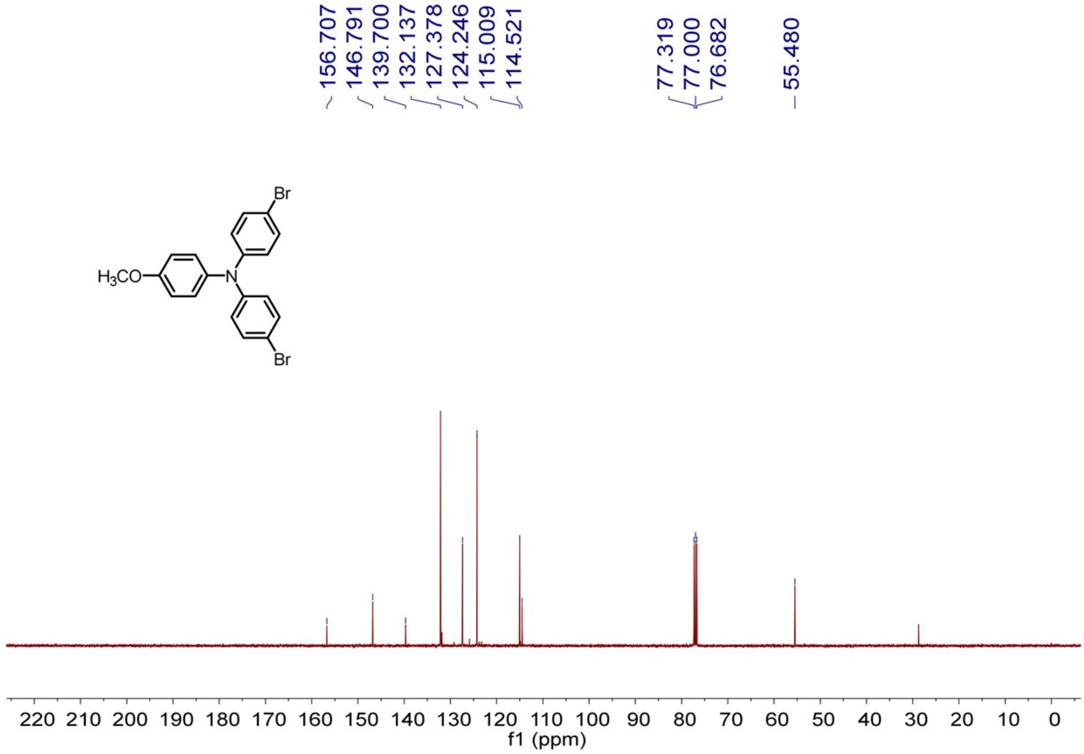

Fig. S6 ^{13}C NMR spectra of **7** (in $CDCl_3$).

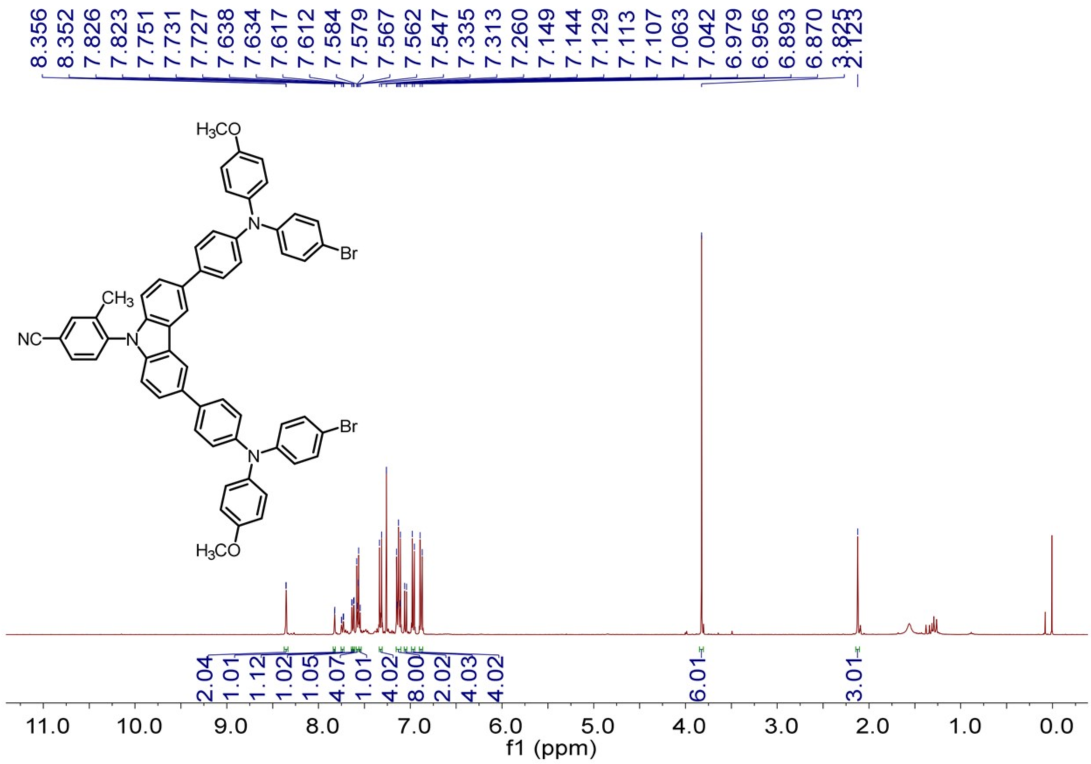

Fig. S7 ^{1}H NMR spectra of **K-2** (in $CDCl_3$).

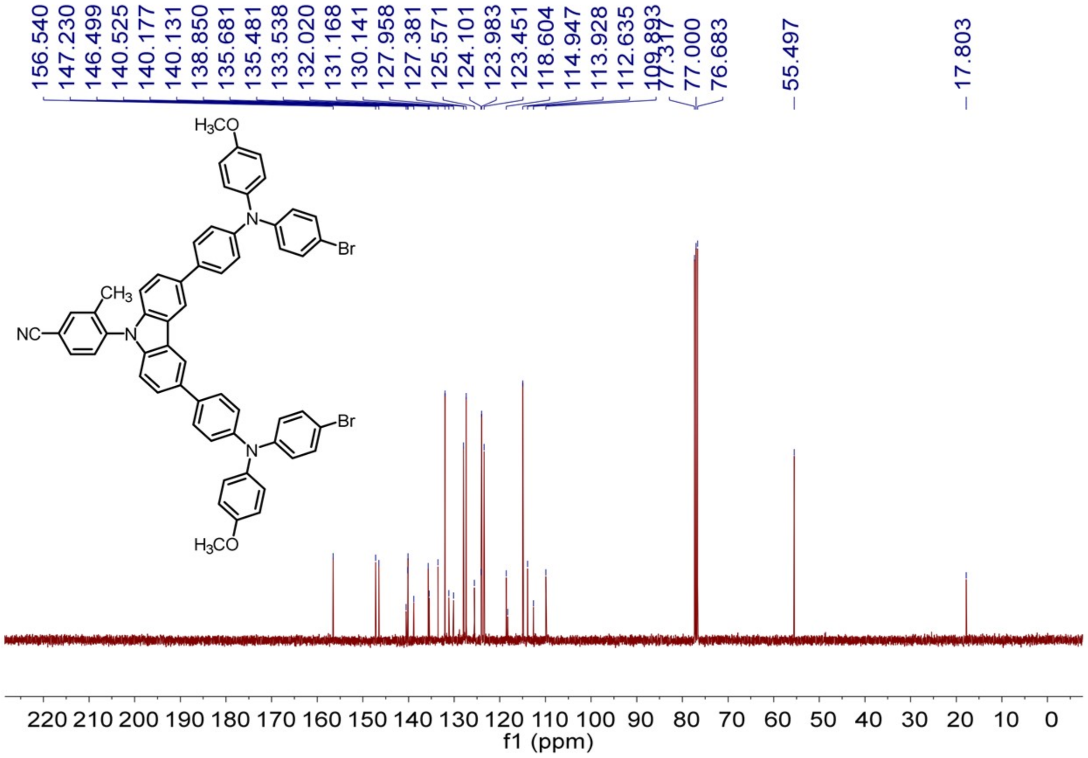

Fig. S8 ^{13}C NMR spectra of **K-2** (in $CDCl_3$).

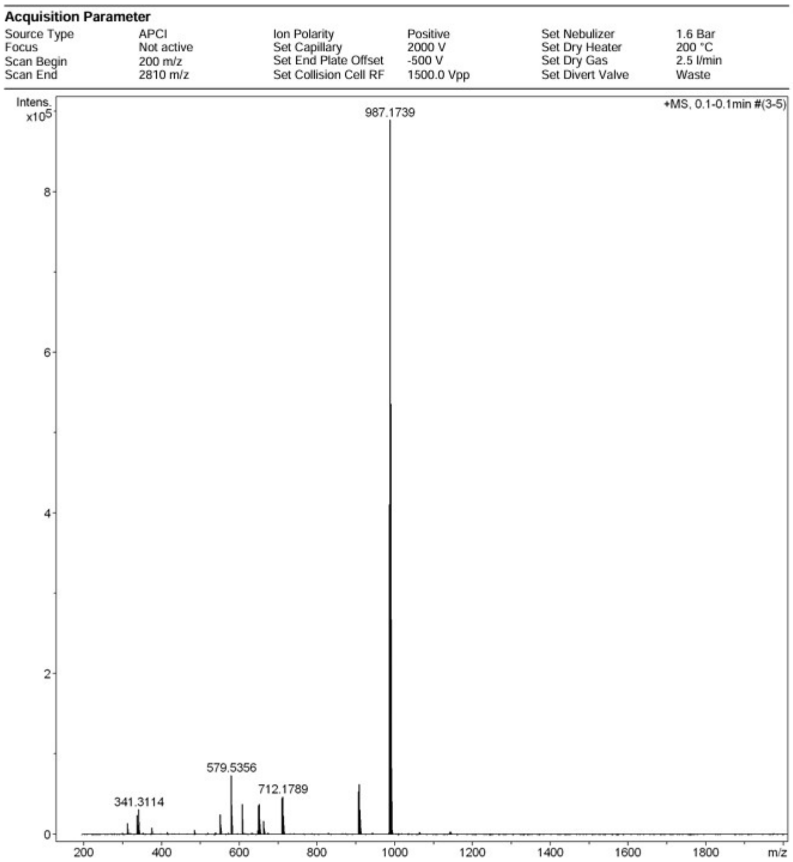

Fig. S9 HRMS spectrum of compound **K-2.**

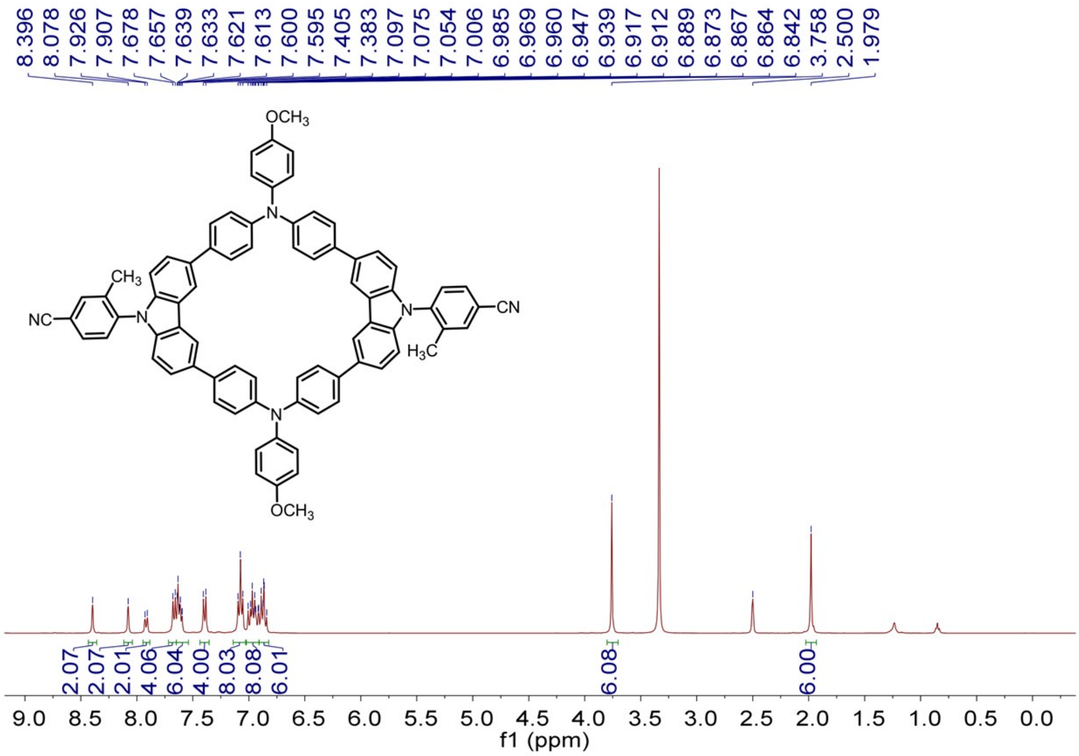

Fig. S10 ^{1}H NMR spectra of **K-1** (DMSO-d_6).

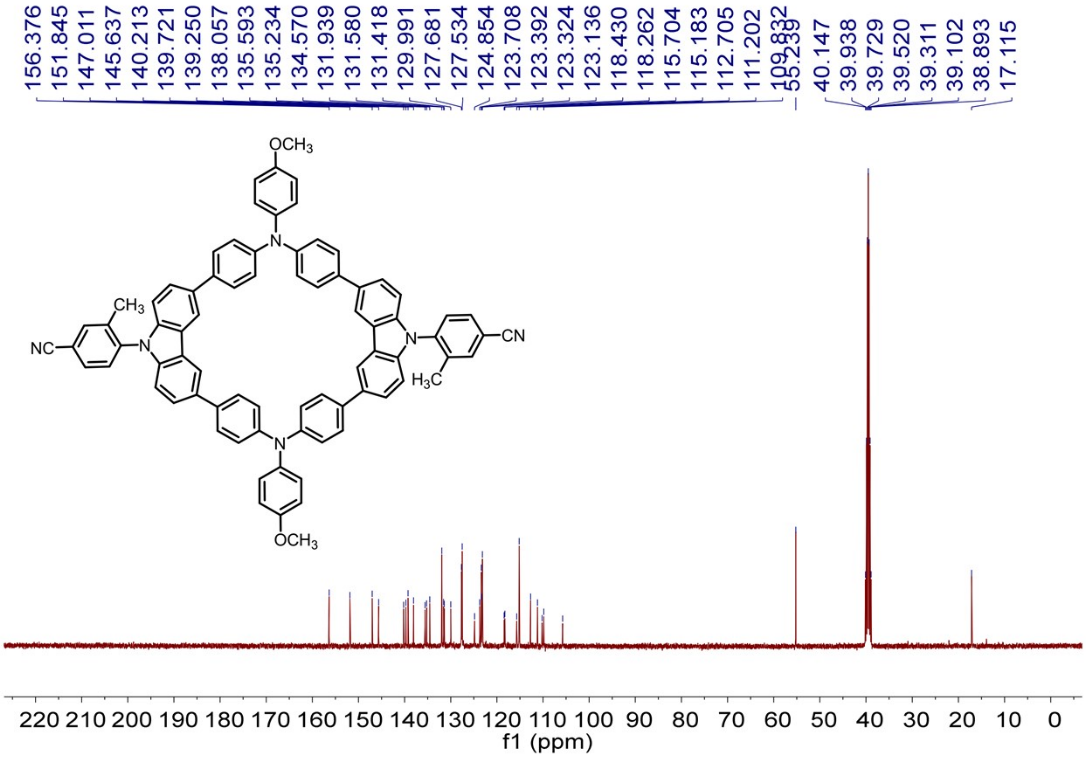

Fig. S11 ^{13}C NMR spectra of **K-1** (DMSO-d_6).

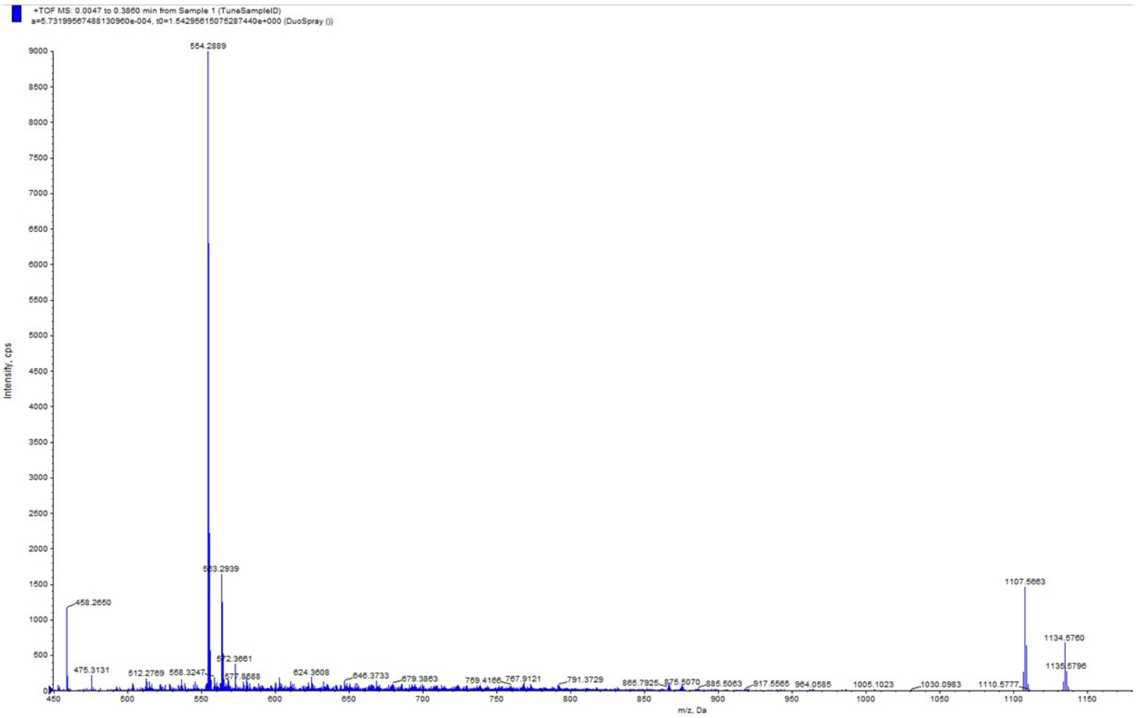

Fig. S12 HRMS spectrum of compound **K-1.**

3. Photophysical spectra

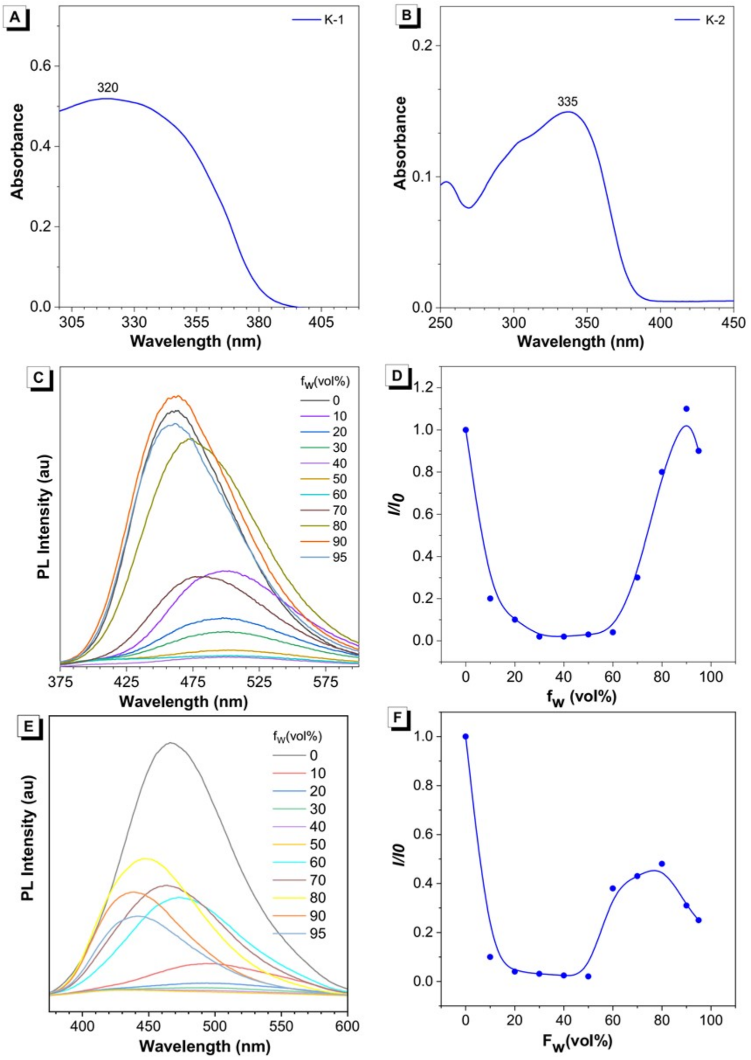

Fig. S13 (A) Absorption spectra of **K-1** in THF, $c = 1 \times 10^{-5}$ M, λ_{max} = 320 nm. (B) Absorption spectra of **K-2** in THF, $c = 1 \times 10^{-5}$ M, λ_{max} = 335 nm. (C) Fluorescence emission spectra of $c = 1 \times 10^{-5}$ M **K-1** in THF/H_2O with different H_2O fraction, λ_{ex} = 320 nm. (D) Fluorescence intensity of versus H_2O fractions. (E) Fluorescence emission spectra of $c = 1 \times 10^{-5}$ M **K-2** in THF/H_2O with different H_2O fraction, λ_{ex} = 335 nm. (F) Fluorescence intensity of versus H_2O fractions.

4. Energy transfer efficiency (Φ_{ET}) and antenna effect (AE) calculation

(1) Energy transfer efficiency (Φ_{ET}), is the ability to transfer energy from donor to acceptor, that is, the ratio of the fluorescence intensity of the donor in the absence of and presence of the acceptor (I_D and I_{DA}). Φ_{ET} was calculated using Equation **S1**:

$$\Phi_{ET} = 1 - I_{DA}/I_D$$

Where I_{DA} and I_D are the fluorescence intensities at 450 nm of **K-1/PBTB** and **K-1** respectively when excited at 320 nm.

Where I_{DA} and I_D are the fluorescence intensities at 450 nm of **K-2/PBTB** and **K-2** respectively when excited at 335 nm.

(2) Antenna effect (AE), is the ability of the acceptor to harvest energy from the donor. AE was calculated using Equation **S2**:

$$AE = (I_{DA,320} - I_{D,320})/I_{DA,450}$$

Where $I_{DA,320}$ is the fluorescence intensity at Maximum emission wavelength nm of **K-1/PBTB** when indirect excitation of the acceptor at 320 nm, $I_{D,320}$ is the fluorescence intensity at Maximum emission wavelength of **K-1** which is normalized with **K-1/PBTB** at 600 nm. $I_{DA,450}$ is the fluorescence intensity at Maximum emission wavelength of **K-1/PBTB** when direct excitation of the acceptor at 320 nm.

$$AE = (I_{DA,335} - I_{D,335})/I_{DA,450}$$

Where $I_{DA,335}$ is the fluorescence intensity at Maximum emission wavelength nm of **K-2/PBTB** when indirect excitation of the acceptor at 335 nm, $I_{D,335}$ is the fluorescence intensity at Maximum emission wavelength of **K-2** which is normalized with **K-2/PBTB** at 590 nm. $I_{DA,450}$ is the fluorescence intensity at Maximum emission wavelength of **K-2/PBTB** when direct excitation of the acceptor at 335 nm.

Table S1. Fluorescence lifetimes of **K-1** and **K-1/PBTB** in H_2O−THF (19/1; v/v)

	**K-1**	**α_1 (%)**	**K-1/PBTB**	**α_2 (%)**
τ_1	0.94	15.90	0.70	39.85
τ_2	3.95	84.10	2.80	60.15
χ^2	1.13	-	1.09	-

Table S2. Energy transfer efficiency and antenna effect of **K-1/PBTB**.

**Sample** (**K-1**, **PBTB**)	**Concentration, Respectively** (**K-1**, **PBTB**)	**Φ_{ET} (%)**	**AE**
1000:5	1×10^{-5} M, 5×10^{-8}M	11.4	24.7
1000:10	1×10^{-5} M, 1×10^{-7} M	40.1	36.5
1000:15	1×10^{-5} M, 1.5×10^{-7} M	50.0	**43.5**
1000:20	1×10^{-5} M, 2×10^{-7} M	60.8	19.9
1000:25	1×10^{-5} M, 2.5×10^{-7} M	68.7	18.5
1000:30	1×10^{-5} M, 3×10^{-7} M	75.0	16.2
1000:40	1×10^{-5} M, 4×10^{-7} M	**82.6**	13.3

Table S3. Fluorescence lifetimes of **K-2** and **K-2/PBTB** in H_2O−THF (19/1; v/v)

	**K-2**	**α_1 (%)**	**K-2/PBTB**	**α_2 (%)**
τ_1	0.60	20.24	3.11	44.30
τ_2	0.45	79.76	2.30	55.70
χ^2	1.13	-	1.29	-

Table S4. Φ_{ET} and AE calculation of the **K-2**/**PBTB** co-assembled film system.

Sample (K-2, PBTB)	Concentration, Respectively (K-2, PBTB)	Φ_{ET} (%)	AE
1000:5	1×10^{-5} M, 5×10^{-8}M	30.0	37.1
1000:10	1×10^{-5} M, 1×10^{-7} M	43.9	32.0
1000:15	1×10^{-5} M, 1.5×10^{-7} M	56.2	29.6
1000:20	1×10^{-5} M, 2×10^{-7} M	65.6	23.5
1000:25	1×10^{-5} M, 2.5×10^{-7} M	69.1	22.6
1000:30	1×10^{-5} M, 3×10^{-7} M	70.0	25.1
1000:40	1×10^{-5} M, 4×10^{-7} M	**77.9**	**37.2**

Table S5. Fluorescence lifetimes of **K-1**/**PBTB** and **K-1**/**PBTB**/**Z1** in H_2O-THF (19/1; v/v).

	**K-1/PBTB**	**α_1 (%)**	**K-1/PBTB/Z1**	**α_2 (%)**
τ_1	3.63	55.88	2.36	60.44
τ_2	11.22	44.12	7.96	39.56
χ^2	1.29	-	1.26	-

Table S6. Fluorescence lifetimes of **K-2**/**PBTB** and **K-2**/**PBTB**/**Z**1 in H_2O-THF (19/1; v/v).

	**K-2/PBTB**	**α_1 (%)**	**K-2/PBTB/Z1**	**α_2 (%)**
τ_1	1.19	9.38	0.84	18.53
τ_2	4.52	59.49	3.31	56.84
χ^2	1.02	-	1.11	-

Table S7. Energy transfer efficiency and antenna effect of **K-1**/**PBTB**/**Z1**

**Sample** (**K-1**, **PBTB**, **Z1**)	**Concentration, Respectively** (**K-1**, **PBTB**, **Z1**)	Φ_{ET} **(%)**	**AE**
1000:40:2	1×10^{-5} M, 4×10^{-7} M, 2×10^{-8} M	27.6	16.8
1000:40:4	1×10^{-5} M, 4×10^{-7} M, 4×10^{-8} M	33.0	18.4
1000:40:6	1×10^{-5} M, 4×10^{-7} M, 6×10^{-8} M	47.2	17.4
1000:40:8	1×10^{-5} M, 4×10^{-7} M, 8×10^{-8} M	49.4	**18.8**
1000:40:10	1×10^{-5} M, 4×10^{-7} M, 1×10^{-7} M	62.1	16.0
1000:40:12	1×10^{-5} M, 4×10^{-7} M, 1.2×10^{-7} M	62.6	17.1
1000:40:14	1×10^{-5} M, 4×10^{-7} M, 1.4×10^{-7} M	**66.4**	18.2

Table S8. Energy transfer efficiency and antenna effect of K-2/PBTB/Z1.

**Sample** (**K-2**, **PBTB**, **Z1**)	**Concentration, Respectively** (**K-2**, **PBTB**, **Z1**)	Φ_{ET} **(%)**	**AE**
1000:40:2	1×10^{-5} M, 4×10^{-7} M, 2×10^{-8} M	24.7	11.4
1000:40:4	1×10^{-5} M, 4×10^{-7} M, 4×10^{-8} M	38.4	11.7
1000:40:6	1×10^{-5} M, 4×10^{-7} M, 6×10^{-8} M	40.1	6.8
1000:40:8	1×10^{-5} M, 4×10^{-7} M, 8×10^{-8} M	47.2	12.6
1000:40:10	1×10^{-5} M, 4×10^{-7} M, 1×10^{-7} M	47.6	10.3
1000:40:12	1×10^{-5} M, 4×10^{-7} M, 1.2×10^{-7} M	59.6	9.1
1000:40:14	1×10^{-5} M, 4×10^{-7} M, 1.4×10^{-7} M	**60.4**	**20.8**

5. Control experiment

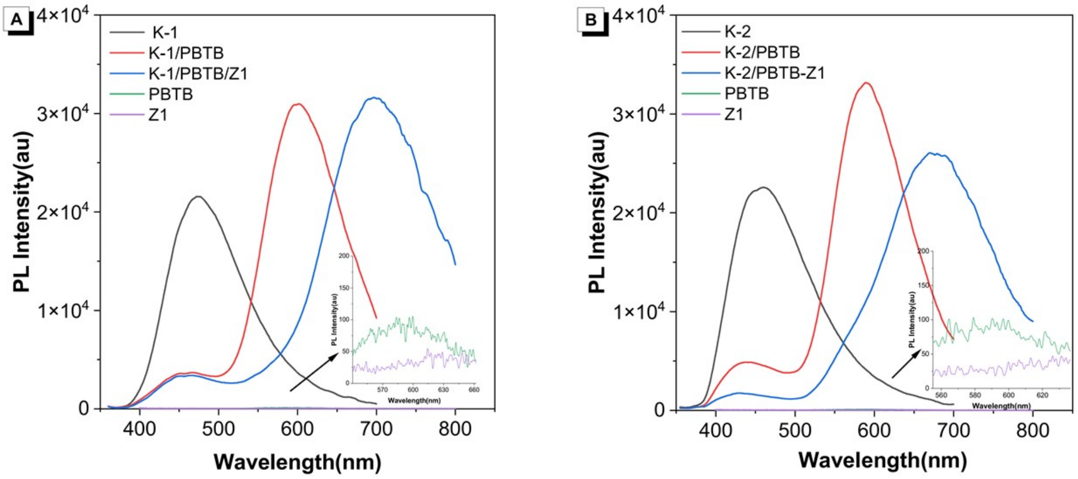

Fig. S14 (A) Fluorescence spectra of **K-1**, **K-1/PBTB**, **K-1/PBTB/Z1**, **PBTB** and **Z1** (λ_{ex} = 320 nm) in THF-H_2O (19:1; v/v). (B) Fluorescence spectra of **K-2**, **K-2/PBTB**, **K-2/PBTB/Z1**, **PBTB** and **Z1** (λ_{ex} = 335 nm) in THF-H_2O (19:1; v/v).

6. SEM and DLS

K-1 **K-2** **PBTB** **Z1**

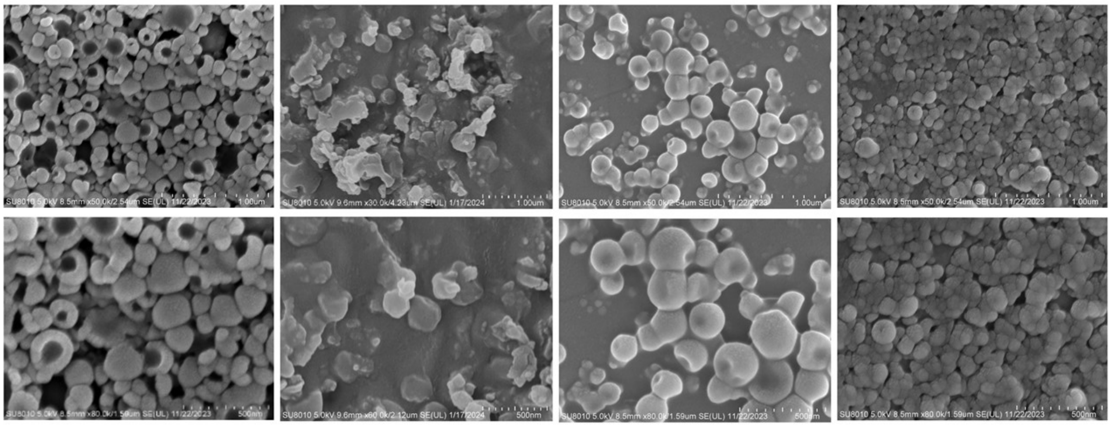

Fig. S15 SEM images of **K-1**, **K-2, PBTB, Z1** in H_2O-THF. (v/v = 19:1).

K-1/PBTB **K-2/PBTB** **K-1/PBTB/Z1** **K-2/PBTB/Z1**

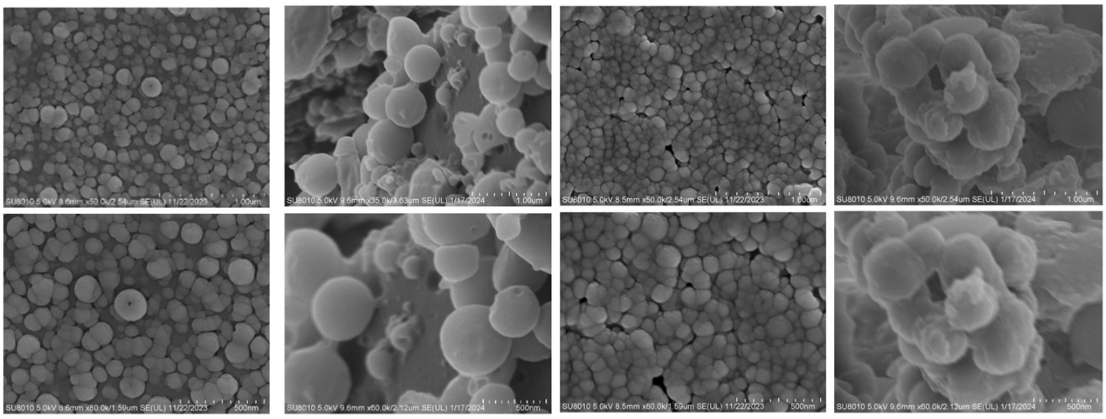

Fig. S16 SEM images of **K-1/PBTB** assembly, **K-2/PBTB** assembly, **K-1/PBTB/Z1** assembly, **K-2/PBTB/Z1** assembly in H_2O-THF. (v/v = 19:1).

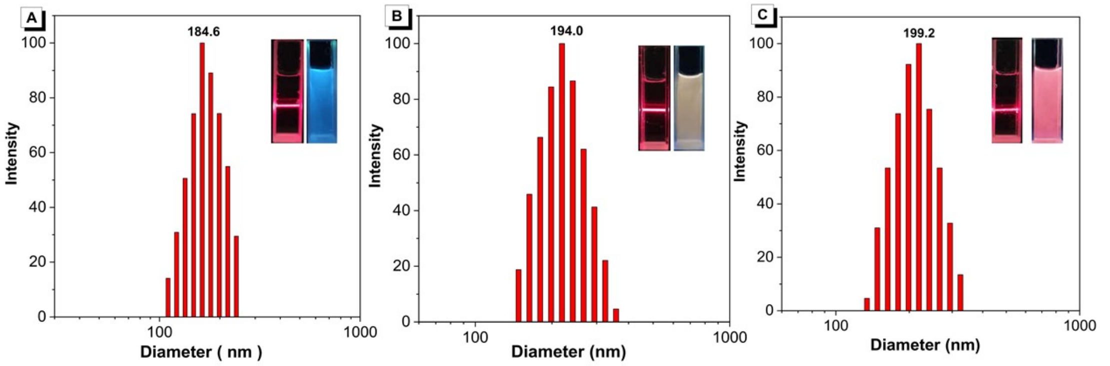

Fig. S17 (A) DLS data of **K-1** NPs, inset: Tyndall effect and fluorescence photograph of **K-1**. (B) DLS data of **K-1/PBTB** NPs, inset: Tyndall effect and fluorescence photograph of **K-1/PBTB**. (C) DLS data of **K-1/PBTB/Z1** NPs, inset: Tyndall effect and fluorescence photograph of **K-1/PBTB/Z1**.

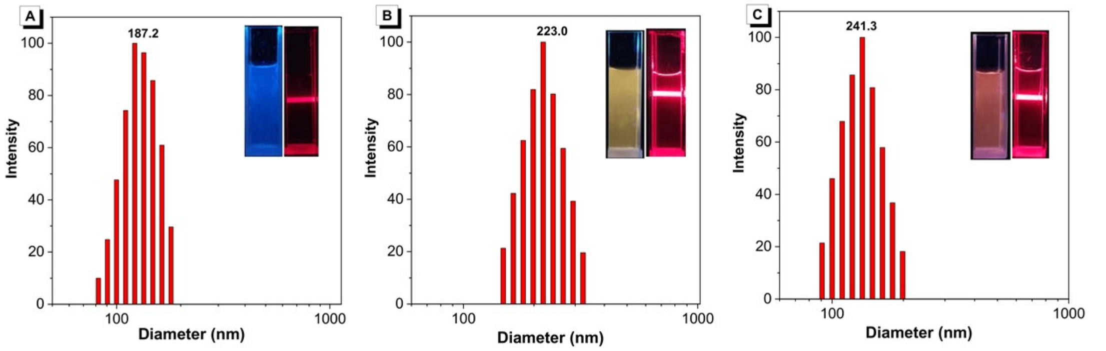

Fig. S18 (A) DLS data of **K-2** NPs, inset: Tyndall effect and fluorescence photograph of **K-2**. (B) DLS data of **K-2**/**PBTB** NPs, inset: Tyndall effect and fluorescence photograph of **K-2**/**PBTB**. (C) DLS data of **K-2**/**PBTB**/**Z1** NPs, inset: Tyndall effect and fluorescence photograph of **K-2**/**PBTB**/**Z1**.

7. Additional Spectra

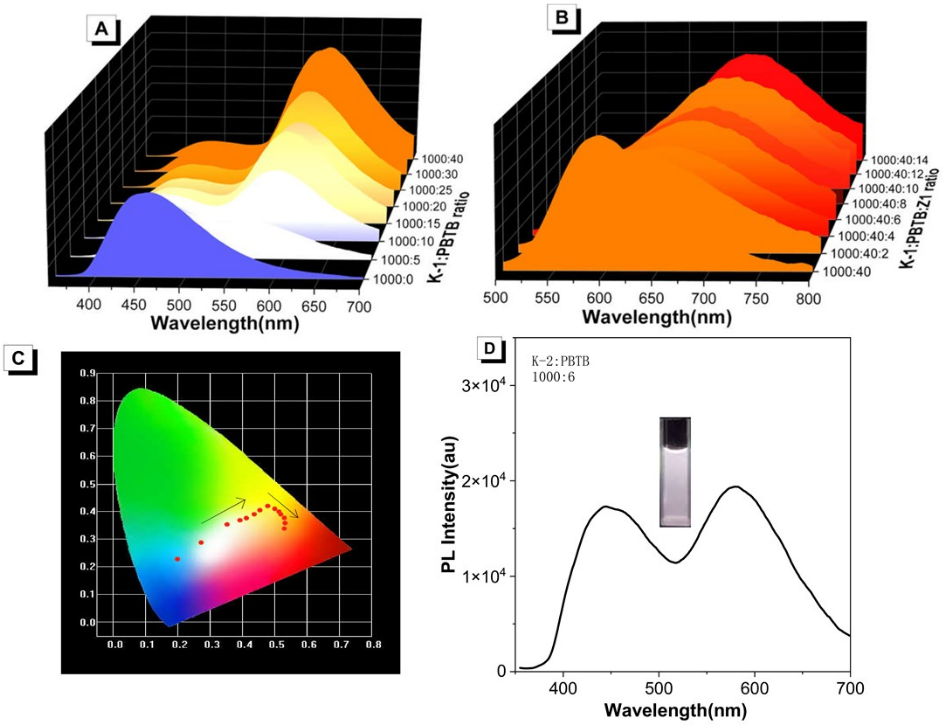

Fig. S19 (A) The fluorescence spectra of **K-2** in water with different concentrations of P BTB. (B). The fluorescence spectra of **K-2/PBTB** in water with different concentrations of **Z1**. (C) CIE chromaticity coordinates of **K-2/PBTB** with different concentrations of **Z1**. (D) Fluorescence spectrum of the white-light emission coordinate (D/A = 500:3) inset: phot ograph of the white-light emission.

8. Photocatalysis

8.1 reaction setup

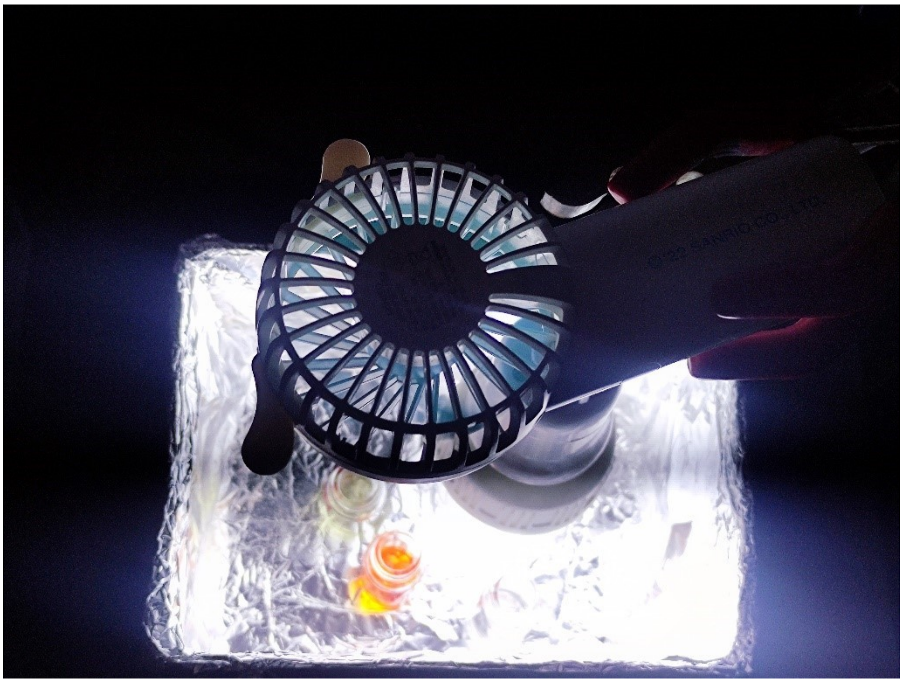

Fig. S20 Typical experimental setup for photoredox catalytic reactions.

8.2 C(sp^2)-P formation

8.2.1 General procedure for C(sp^2)-P formation

Diphenylphosphine oxide **9** (224.6 mg, 1.2 mmol) and **K-1** (5 mol%) was added to a tube and equipped with a stir bar. Then, benzothiazole **8a** (22 uL, 0.2 mmol), H_2O (1.9 mL), PBTB (40 uL, 1×10^{-5} M) and THF (40 mL) was injected in the reaction tube with magnetic stirring. The reaction mixture was stirred and irradiated by banded 30 W white LED under air at room temperature for 24 h. The resulting mixture was extracted with ethyl acetate, washed with saturated $NaHCO_3$ solution and brine. The organic layer was collected and concentrated under reduced pressure. The residue was purified by column chromatography on silica gel using petroleum ether/ethyl acetate as eluent (PE/EtOAc = 4:1 to 2:1).

8.2 Mechanism study

8.2.1 Control experiment performed with TEMPO

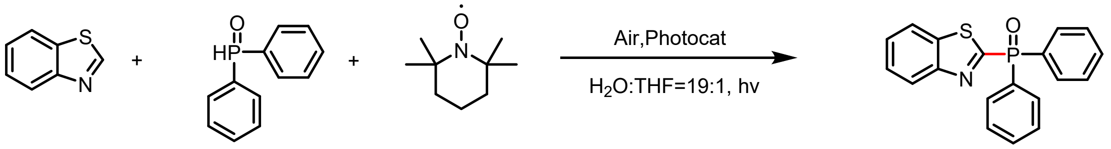

8a **9** **TEMPO** **10a** (trace)

Diphenylphosphine oxide **9** (224.6 mg, 1.2 mmol) and **K-1** (5 mol%) was added to a tube equipped with a stir bar. Then, benzothiazole derivatives (22 uL, 0.1 mmol), H_2O (1.9 mL) PBTB (40 uL, 1×10^{-5} M), THF (40 uL) and TEMPO (0.3 mmol) was injected in the reaction tube with magnetic stirring. The reaction mixture was stirred and irradiated by banded white LED under air at room temperature for 24 h. Only trace amounts of the product **10a** can be observed.

8.2.2 Control experiment performed with BHT

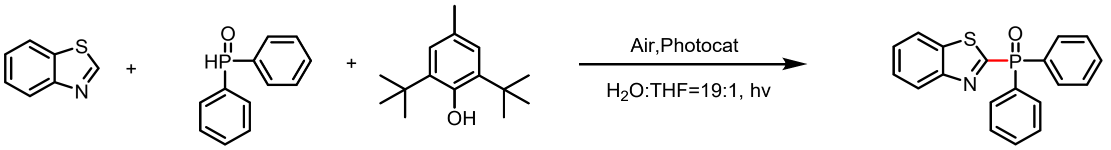

8a **9** **BHT** **10a** (trace)

Diphenylphosphine oxide **9** (224.6 mg, 1.2 mmol) and **K-1** (5 mol%) was added to a tube equipped with a stir bar. Then, benzothiazole **8a** (22 uL, 0.1 mmol), H_2O (1.9 mL), **PBTB** (40 uL, 1×10^{-5} M), THF (40 uL) and BHT (0.3 mmol) was injected in the reaction tube with magnetic stirring. The reaction mixture was stirred and irradiated by banded white LED under air at room temperature for 24 h. Only trace amounts of the product **10a** can be observed.

8.3 Substrate scope of the C(sp^2)–P bonds formation reaction

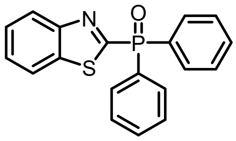

^{1}H NMR (400 MHz, $CDCl_3$) δ = 8.20 (d, J = 7.6 Hz, 1H), 8.02-7.94 (m, 5H), 7.59-7.47 (m, 8H). ^{13}C NMR (100 MHz, $CDCl_3$) δ = 166.78 (d, J = 126.3 Hz), 155.34 (d, J = 21.5 Hz), 136.79, 132.58 (d, J = 2.8 Hz), 131.90 (d, J = 10.2 Hz), 130.49 (d, J = 108.3 Hz), 128.61 (d, J = 12.8 Hz), 126.63, 126.58, 124.73, 122.06. ^{31}P NMR (162 MHz, $CDCl_3$) δ = 19.96.

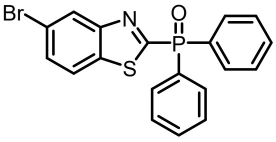

^{1}H NMR (400 MHz, $CDCl_3$) δ = 8.34 (d, J = 0.5 Hz, 1H), 7.99-7.93 (m, 4H), 7.86 (d, J = 8.4 Hz, 1H), 7.59-7.56 (m, 3H), 7.52-7.48 m, 4H). ^{13}C NMR (100 MHz, $CDCl_3$) δ = 169.05 (d, J = 123.4 Hz), 156.39 (d, J = 21.3 Hz), 135.54, 132.71 (d, J = 2.9 Hz), 131.85 (d, J = 10.1 Hz), 130.66 (d, J = 108.5 Hz), 129.76, 128.66 (d, J = 12.9 Hz), 127.47, 123.09, 120.36. ^{31}P NMR (162 MHz, $CDCl_3$) δ = 19.69.

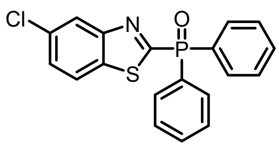

^{1}H NMR (400 MHz, $CDCl_3$) δ = 8.16 (s, 1H), 7.98-7.93 (m, 4H), 7.90 (d, J = 8.4 Hz, 1H), 7.58-7.55 (m, 2H), 7.51-7.46 (m, 4H), 7.44 (d, J = 2.2 Hz, 1H). ^{13}C NMR (100 MHz, $CDCl_3$) δ = 169.21 (d, J = 123.5 Hz), 156.03 (d, J = 21.3 Hz), 134.98, 132.78, 132.67 (d, J = 2.8 Hz), 131.81 (d, J = 10.1 Hz), 130.65 (d, J = 108.5 Hz), 128.62 (d, J = 12.8 Hz), 127.15, 124.30, 122.75. ^{31}P NMR (162 MHz, $CDCl_3$) δ = 19.73.

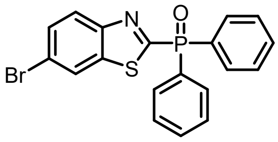

^{1}H NMR (400 MHz, $CDCl_3$) δ = 8.15 (s, 1H), 8.02 (d, J = 8.8 Hz, 1H), 7.96 (dd, J = 12.8, 8.0 Hz, 4H), 7.63 (d, J = 8.8 Hz, 1H), 7.58 (t, J = 7.2 Hz, 2H), 7.52-7.48 (m, 4H). ^{13}C NMR (100 MHz, $CDCl_3$) δ = 167.72 (d, J = 124.1 Hz), 154.14 (d, J = 21.4 Hz), 138.39, 132.71 (d, J = 2.9 Hz), 131.86 (d, J = 10.2 Hz), 130.67 (d, J = 108.5 Hz), 130.29, 128.66 (d, J = 12.7 Hz), 125.72, 124.58, 120.74. ^{31}P NMR (162 MHz, $CDCl_3$) δ = 19.99.

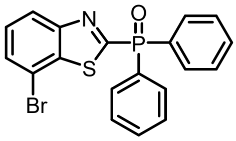

^{1}H NMR (400 MHz, $CDCl_3$) δ = 8.11 (d, J = 8.4 Hz, 1H), 7.96 (dd, J = 12.0, 7.2 Hz, 4H), 7.61-7.56 (m, 3H), 7.52-7.48 (m, 4H), 7.41 (t, J = 8.4 Hz, 1H). ^{13}C NMR (100 MHz, $CDCl_3$) δ = 167.74 (d, J = 122.7 Hz), 154.84 (d, J = 21.2 Hz), 139.88, 132.72 (d, J = 2.8 Hz), 131.90 (d, J = 10.2 Hz), 130.63 (d, J = 108.4 Hz), 129.27, 128.67 (d, J = 12.8 Hz), 127.81, 123.55, 114.34. ^{31}P NMR (162 MHz, $CDCl_3$) δ = 19.98.

^{1}H-NMR, ^{13}C-NMR and ^{31}P-NMR spectra of 10a-10e

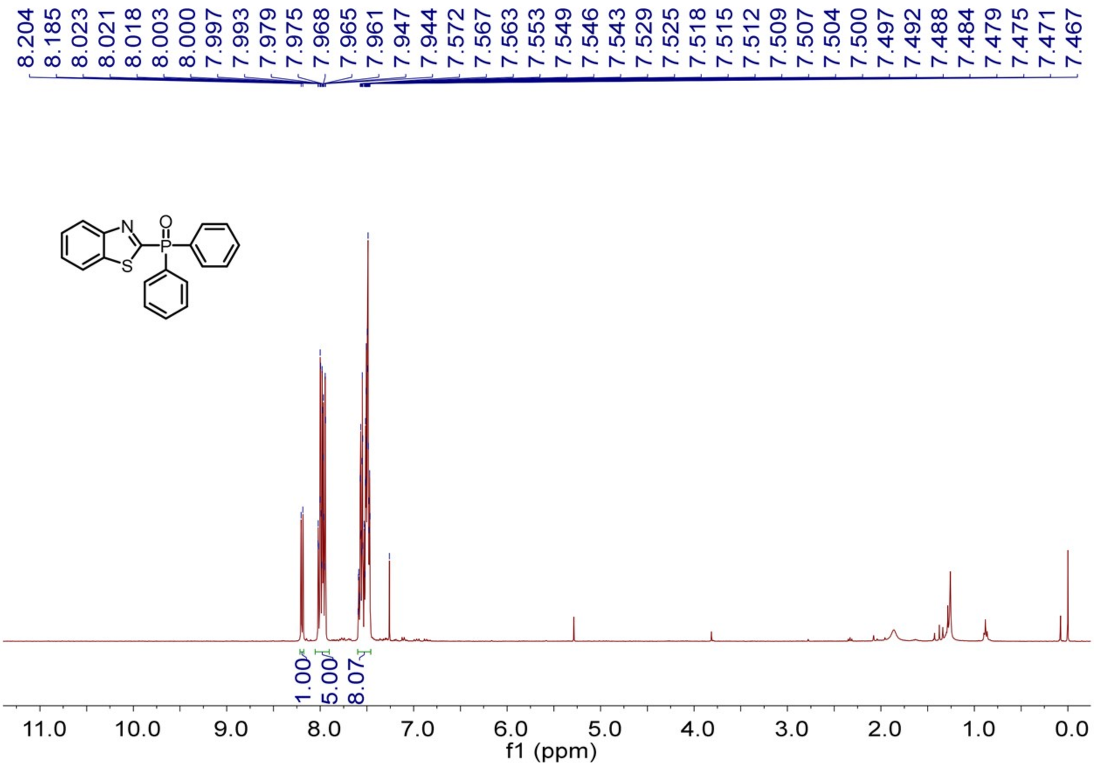

Fig. S21 ^{1}H NMR spectra of **10a** (in $CDCl_3$).

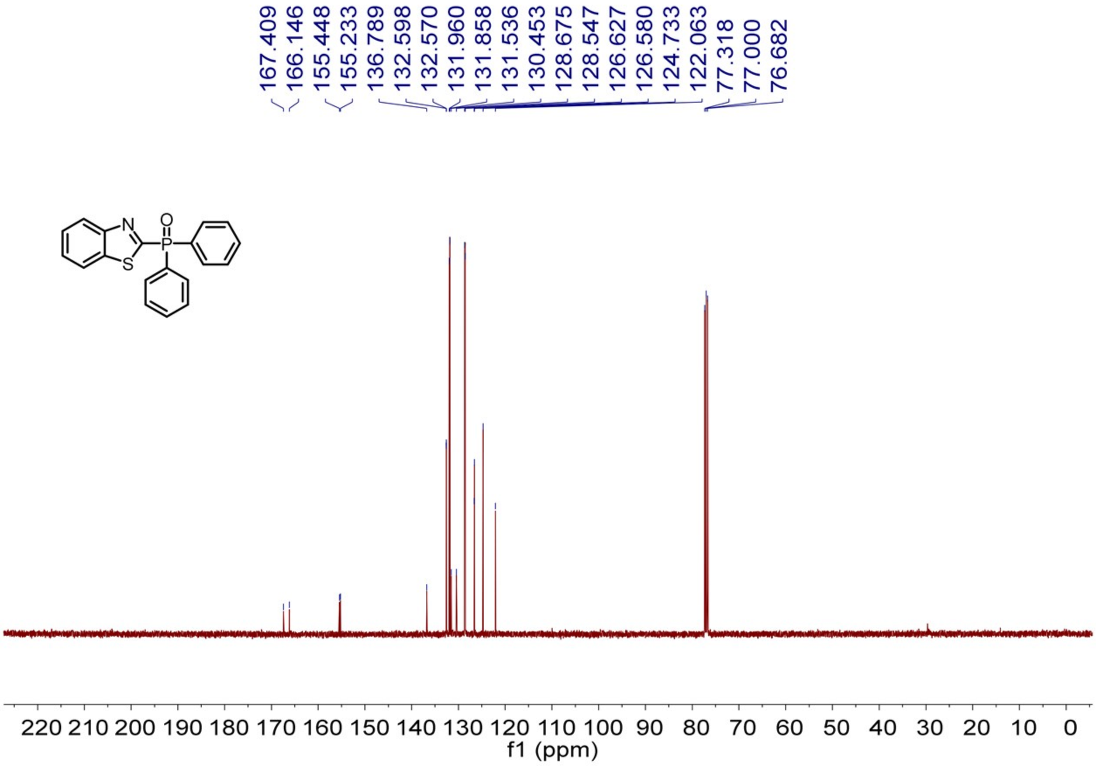

Fig. S22 ^{13}C NMR spectra of **10a** (in $CDCl_3$).

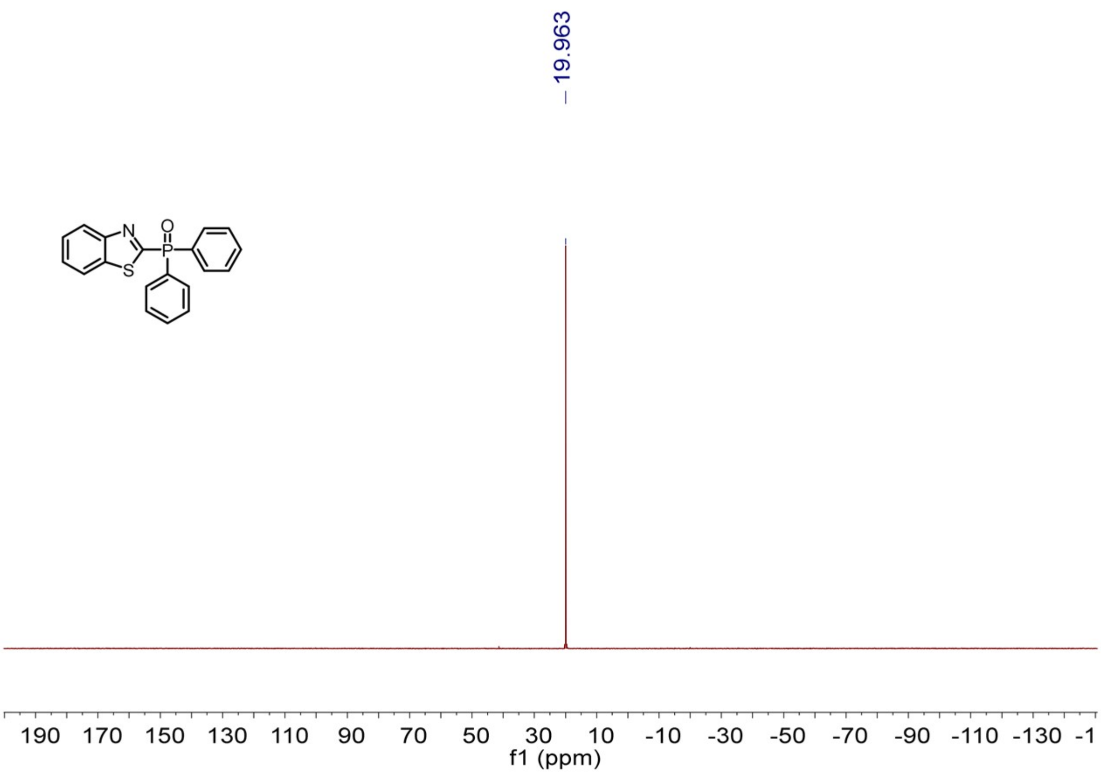

Fig. S23 ^{31}P NMR spectra of **10a** (in $CDCl_3$).

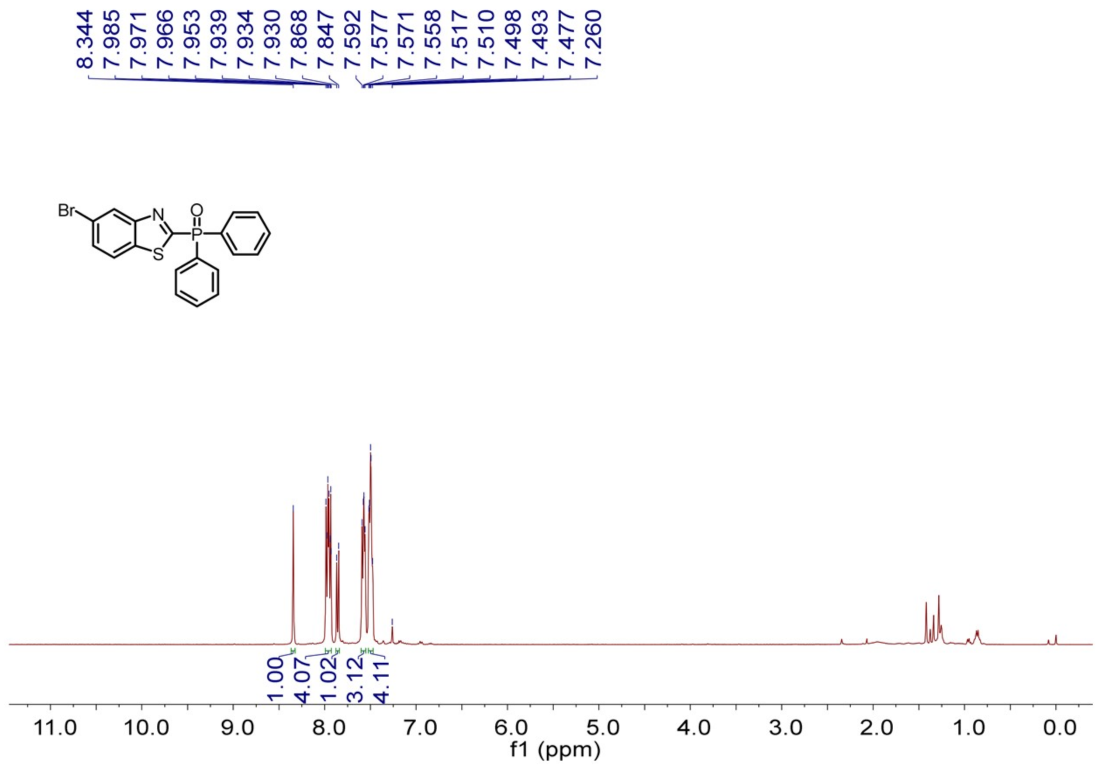

Fig. S24 ^{1}H NMR spectra of **10b** (in $CDCl_3$).

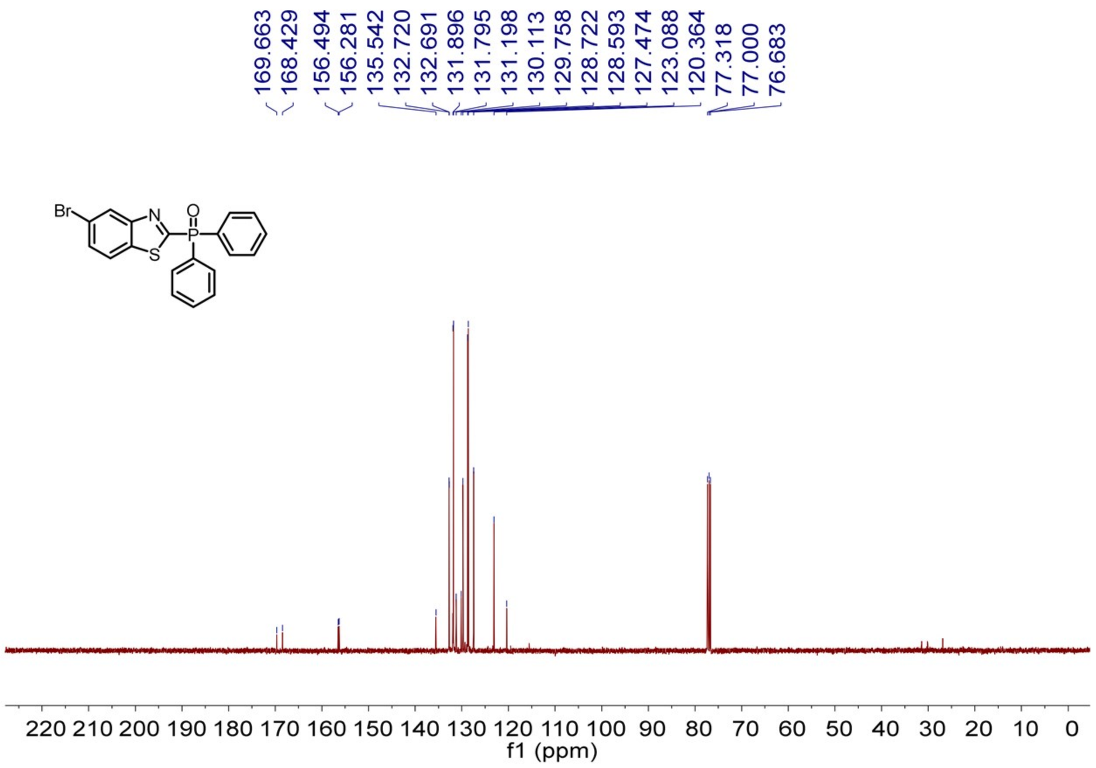

Fig. S25 ^{13}C NMR spectra of **10b** (in $CDCl_3$).

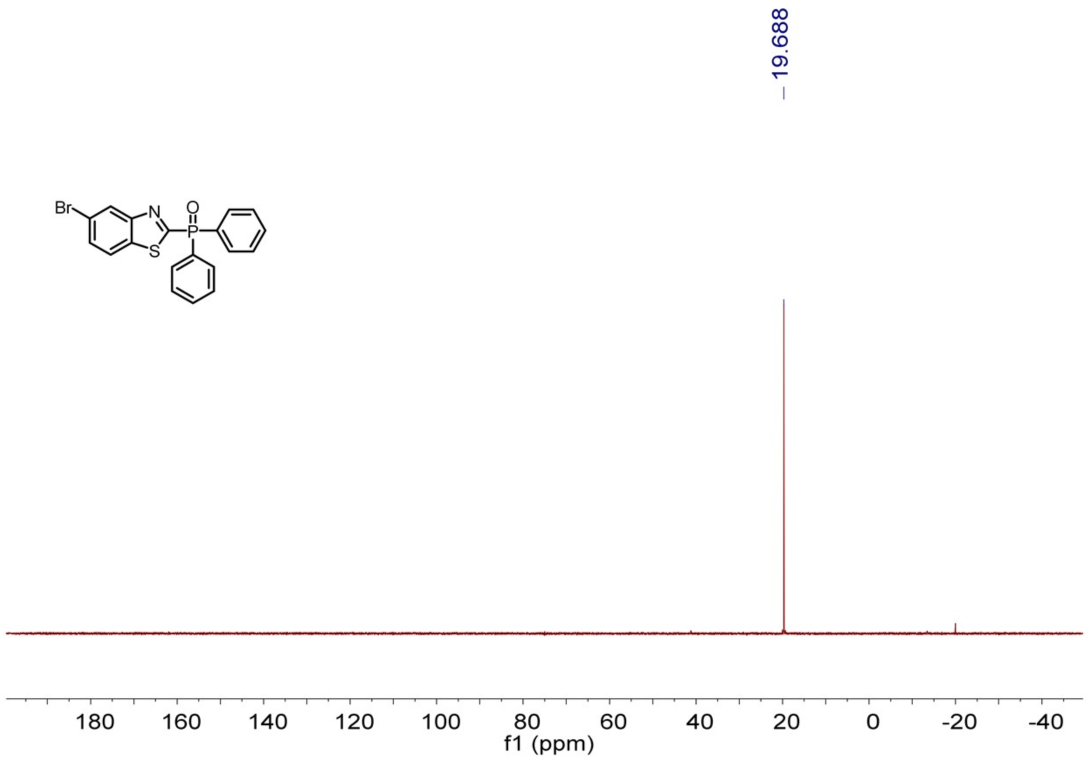

Fig. S26 ^{31}P NMR spectra of **10b** (in $CDCl_3$).

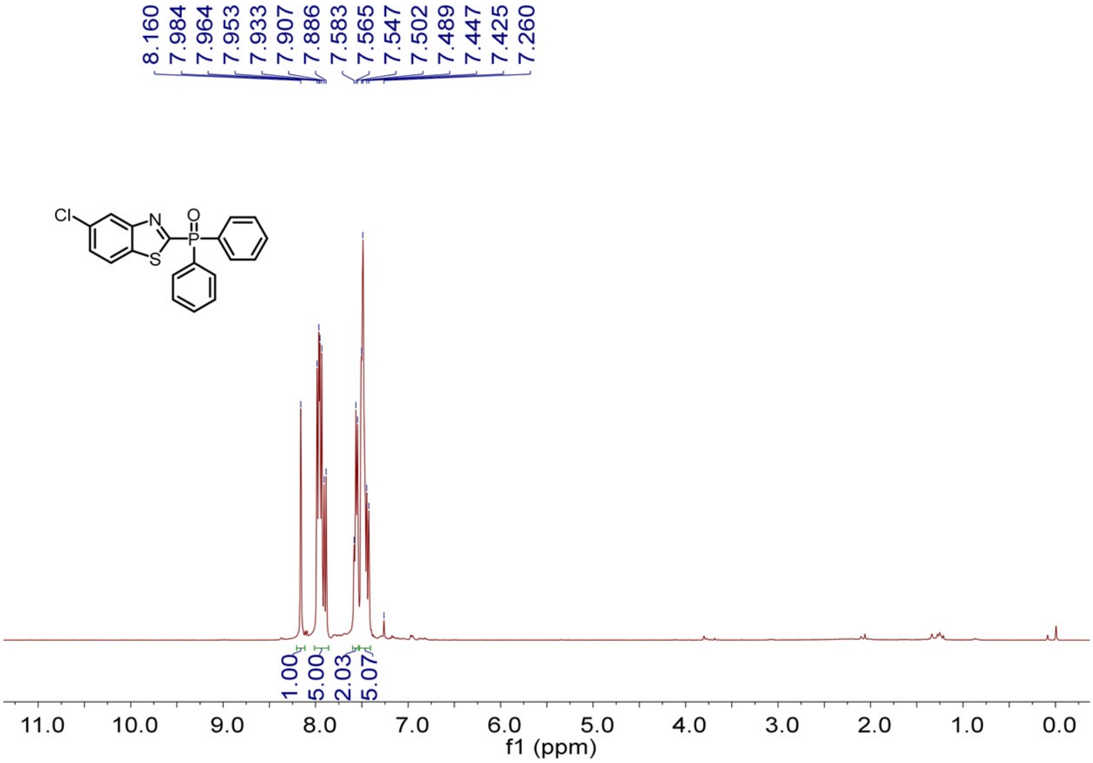

Fig. S27 ^{1}H NMR spectra of **10c** (in $CDCl_3$).

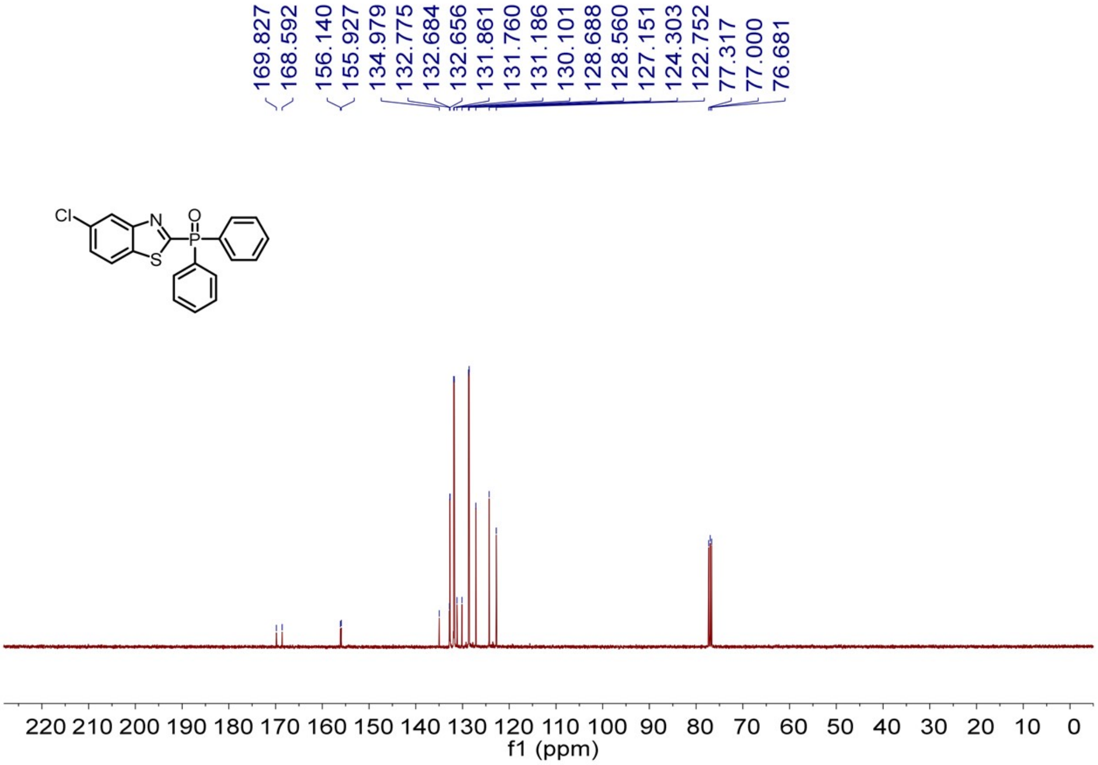

Fig. S28 ^{13}C NMR spectra of **10c** (in $CDCl_3$).

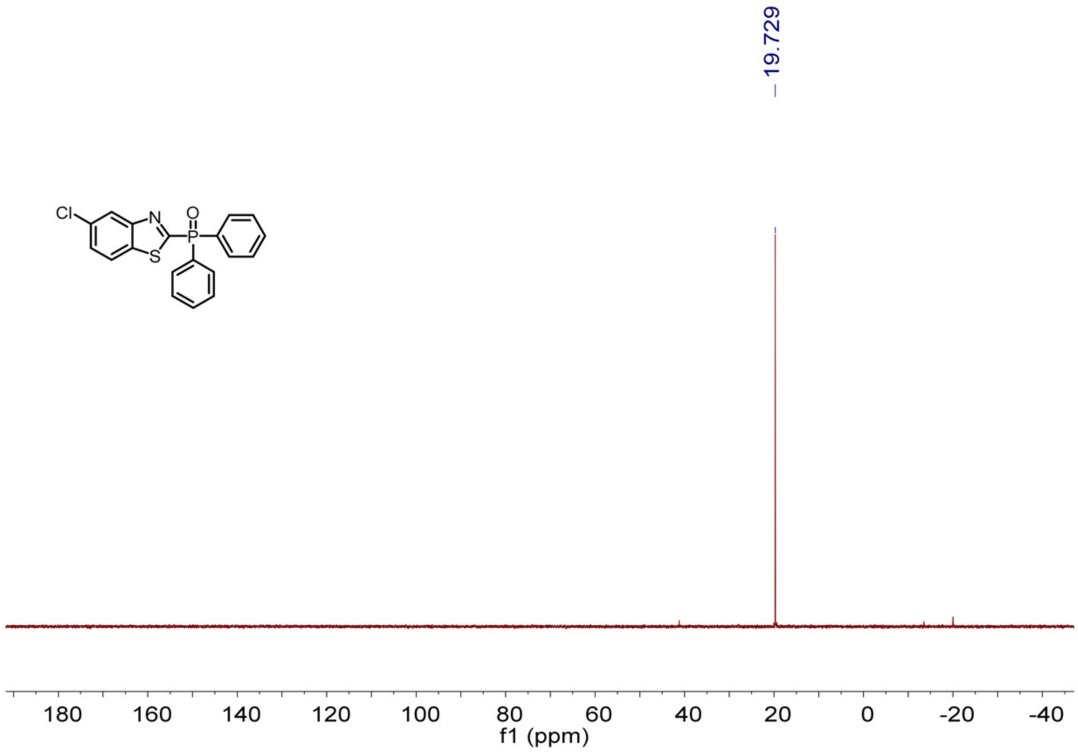

Fig. S29 ^{31}P NMR spectra of **10c** (in $CDCl_3$).

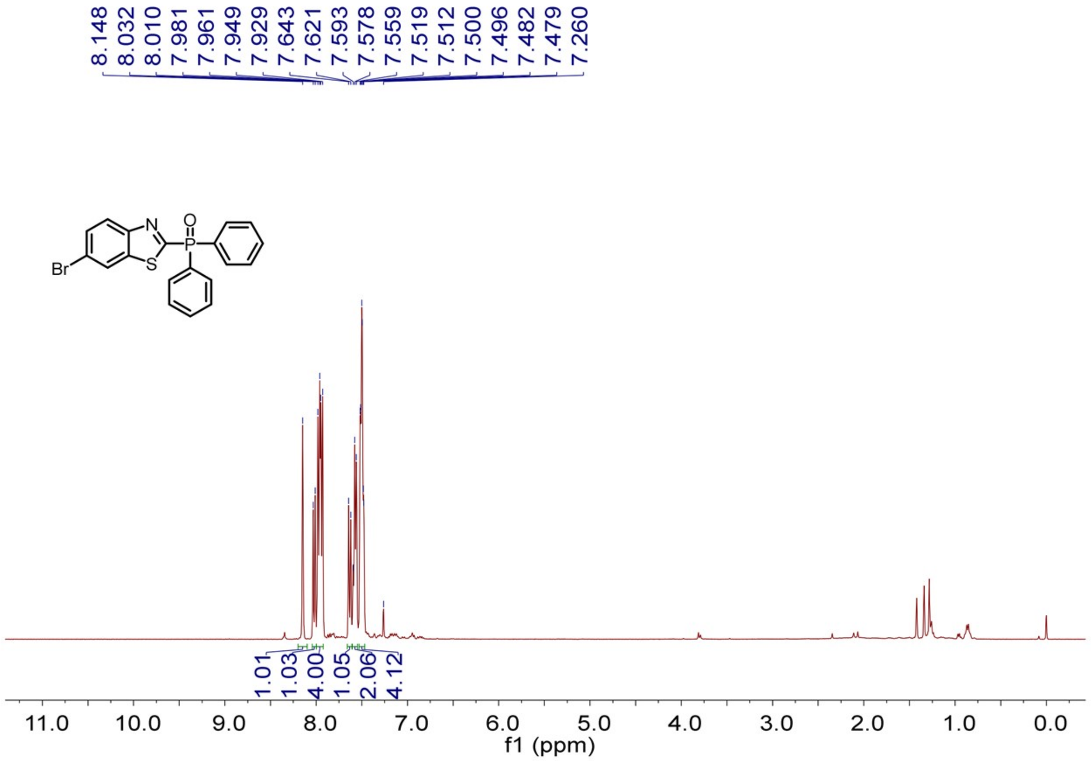

Fig. S30 ^{1}H NMR spectra of **10d** (in $CDCl_3$).

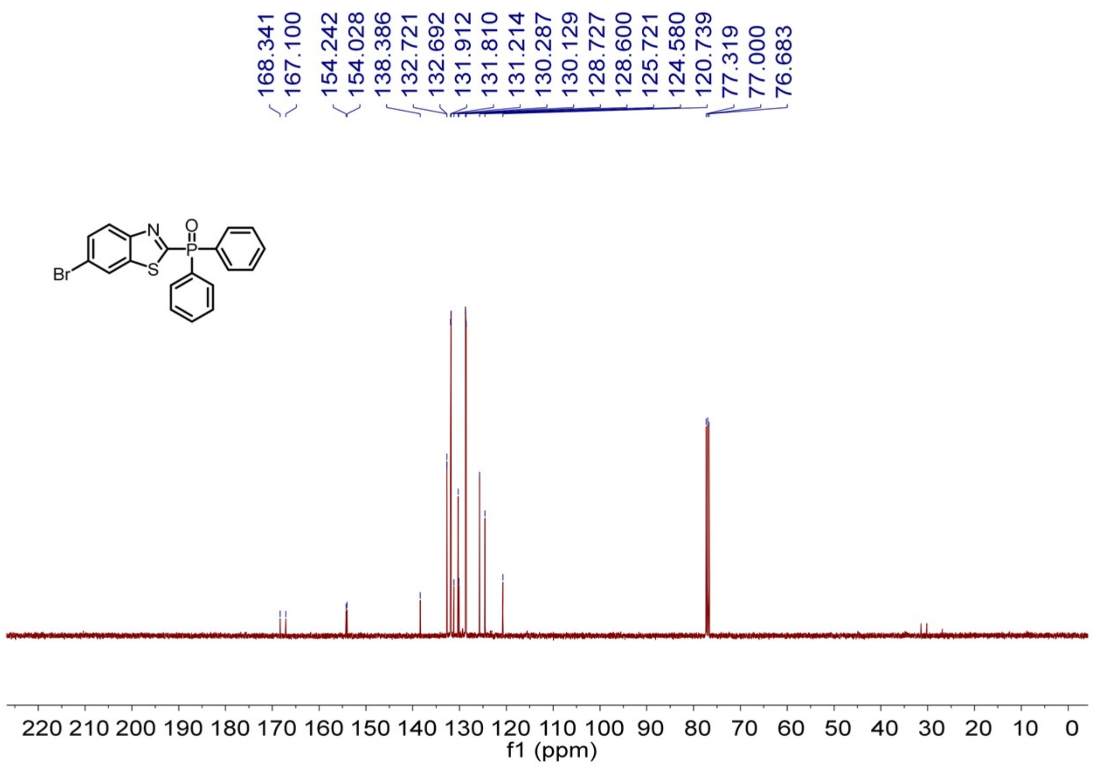

Fig. S31 ^{13}C NMR spectra of **10d** (in $CDCl_3$).

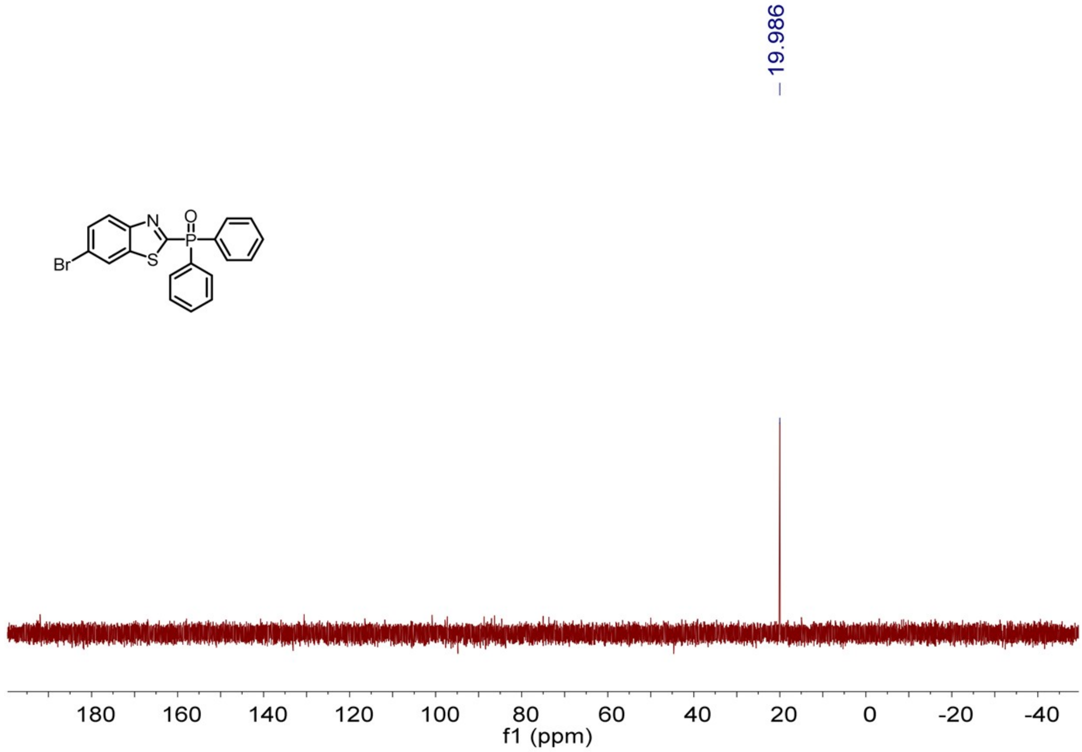

Fig. S32 ^{31}P NMR spectra of **10d** (in $CDCl_3$).

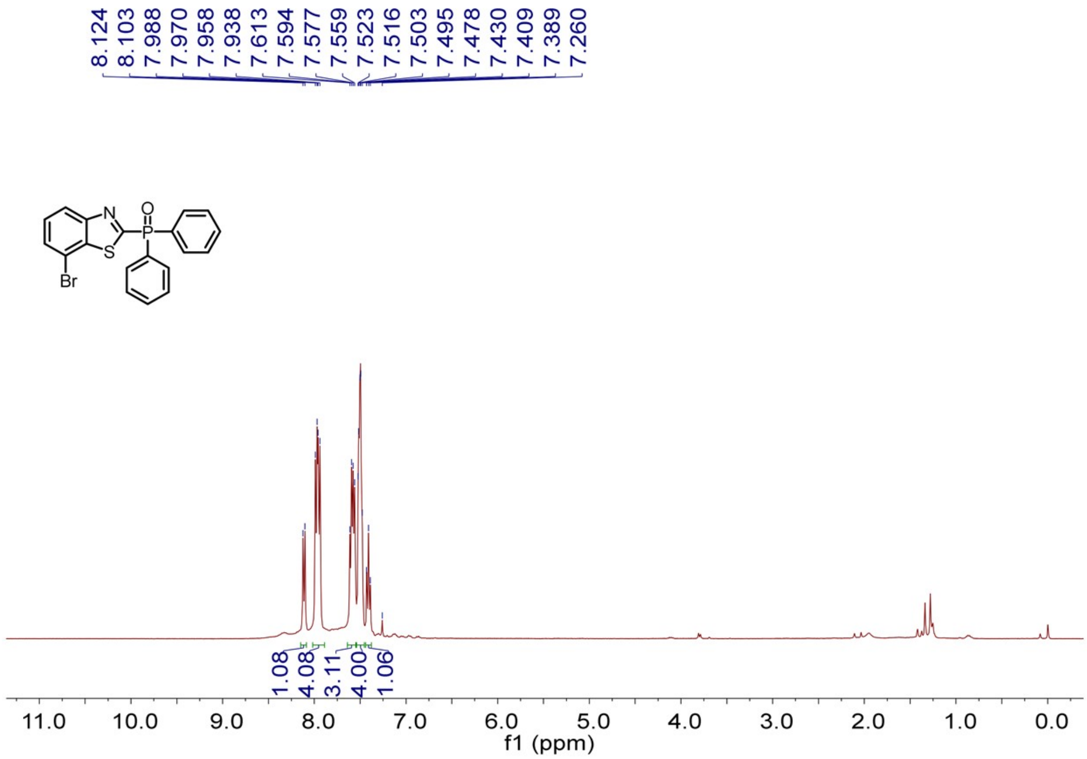

Fig. S33 ^{1}H NMR spectra of **10e** (in $CDCl_3$).

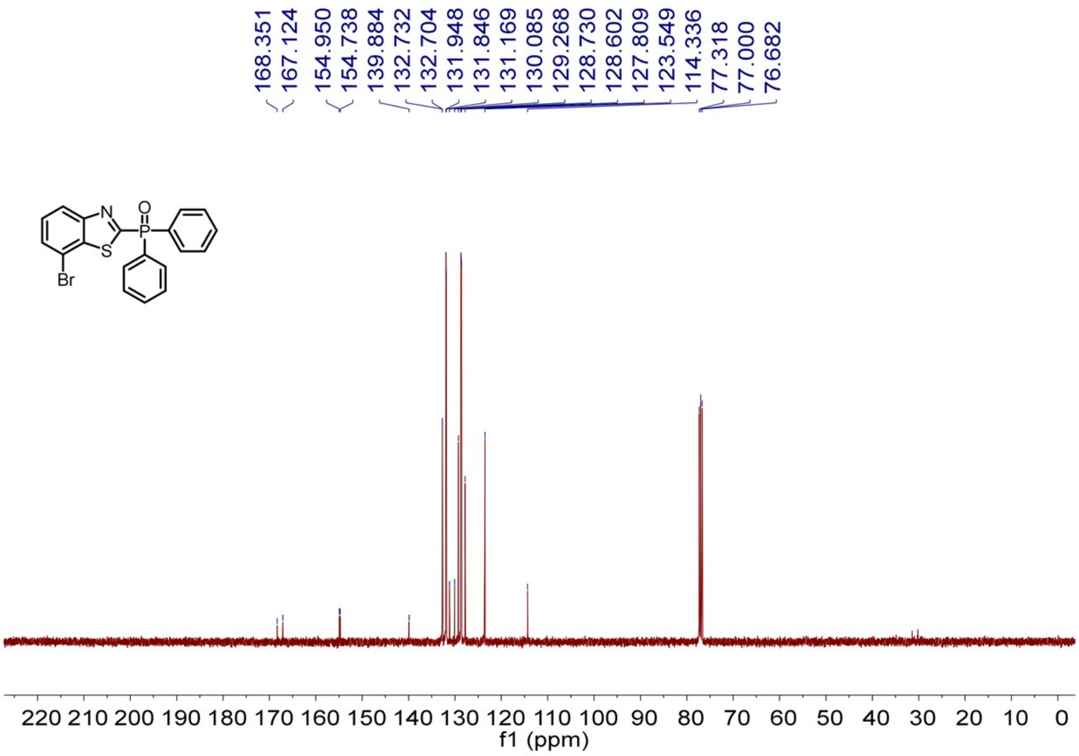

Fig. S34 ^{13}C NMR spectra of **10e** (in $CDCl_3$).

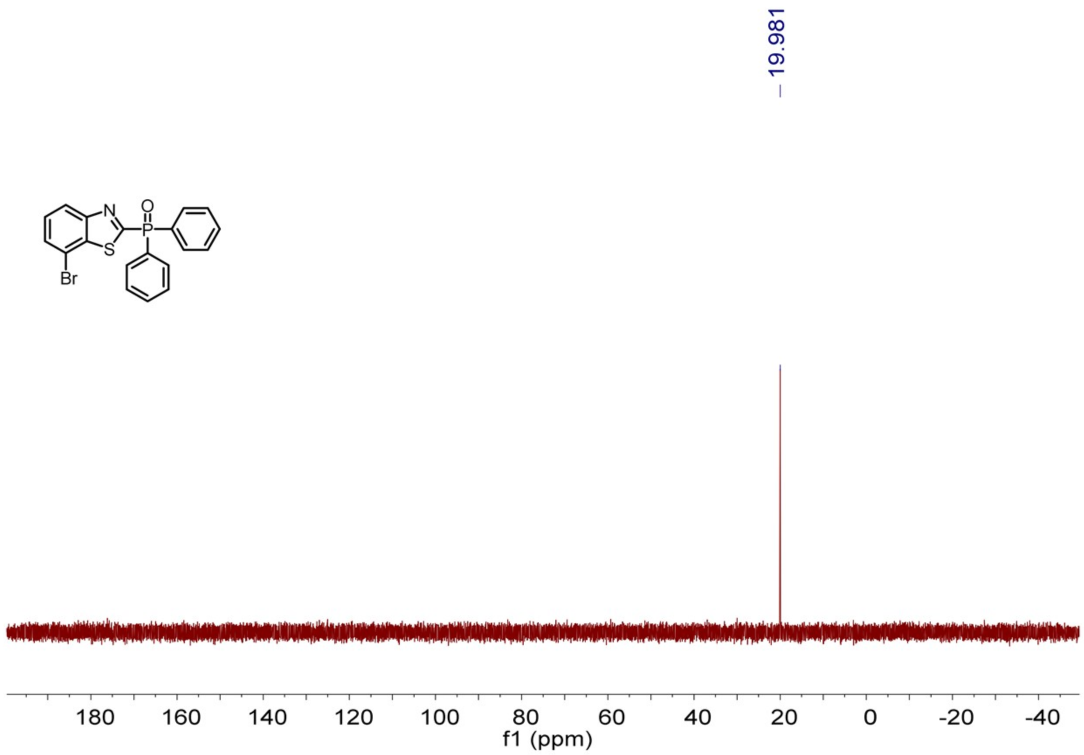

Fig. S35 ^{31}P NMR spectra of **10e** (in $CDCl_3$).

9. References

S1. F.-R. Xu, G.-L. Zhang, K. Zhang, P. Chen, Q. Wang, Y. Pan, B. Z. Tang and H.-T. Feng, *Aggregate*, 2024, **0**, e699. In press, doi: 10.1002/agt2.699.

S2. T. Gallavardin, A. Mulas, L. Norel, S. Rigaut, A. Brosseau, R. Métivier and E. Ishow, *J. Phys. Chem. C*, 2021, **125**, 4665-4674.
